# Revealing Structural Evolution of Nickel Phosphide-Iron Oxide Core–Shell Nanocatalysts in Alkaline Medium for the Oxygen Evolution Reaction

**DOI:** 10.1021/acs.chemmater.4c00379

**Published:** 2024-06-21

**Authors:** Ryan H. Manso, Jiyun Hong, Wei Wang, Prashant Acharya, Adam S. Hoffman, Xiao Tong, Feng Wang, Lauren F. Greenlee, Yimei Zhu, Simon R. Bare, Jingyi Chen

**Affiliations:** † Department of Chemistry and Biochemistry, University of Arkansas, Fayetteville, Arkansas 72701, United States; ‡ Stanford Synchrotron Radiation Lightsource, SLAC National Accelerator Laboratory, Menlo Park, California 94025, United States; § Condensed Matter Physics and Materials Science Department, Brookhaven National Laboratory, Upton, New York 11973, United States; ∥ Ralph E. Martin Department of Chemical Engineering, University of Arkansas, Fayetteville, Arkansas 72701, United States; ⊥ Center for Functional Nanomaterials, Brookhaven National Laboratory, Upton, New York 11973, United States; # Department of Chemical Engineering, Pennsylvania State University, University Park, Pennsylvania 16802, United States

## Abstract

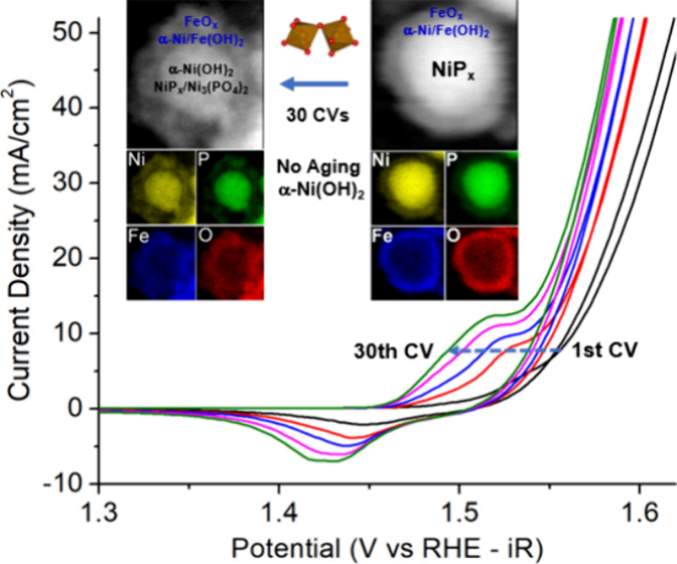

Metal phosphide-containing materials have emerged as a potential candidate of nonprecious metal-based catalysts for alkaline oxygen evolution reaction (OER). While it is known that metal phosphide undergoes structural evolution, considerable debate persists regarding the effects of dynamics on the surface activation and morphological stability of the catalysts. In this study, we synthesize NiP_*x*_-FeO_*x*_ core–shell nanocatalysts with an amorphous NiP_*x*_ core designed for enhanced OER activity. Using *ex situ* X-ray absorption spectroscopy, we elucidate the local structural changes as a function of the cyclic voltammetry cycles. Our studies suggest that the presence of corner-sharing octahedra in the FeO_*x*_ shell improves structural rigidity through interlayer cross-linking, thereby inhibiting the diffusion of OH^–^/H_2_O. Thus, the FeO_*x*_ shell preserves the amorphous NiP_*x*_ core from rapid oxidation to Ni_3_(PO_4_)_2_ and Ni(OH)_2_. On the other hand, the incorporation of Ni from the core into the FeO_*x*_ shell facilitates absorption of hydroxide ions for OER. As a result, Ni/Fe(OH)_*x*_ at the surface oxidizes to the active γ-(oxy)hydroxide phase under the applied potentials, promoting OER. This intriguing synergistic behavior holds significance as such a synthetic route involving the FeO_*x*_ shell can be extended to other systems, enabling manipulation of surface adsorption and diffusion of hydroxide ions. These findings also demonstrate that nanomaterials with core–shell morphologies can be tuned to leverage the strength of each metallic component for improved electrochemical activities.

## Introduction

Water electrolysis under alkaline conditions has drawn increased attention in recent years because it holds promise to generate clean H_2_ at a low cost.^1,2^ The efficiency of this process is not yet optimal. One of the main obstacles is the sluggish kinetics of the oxygen evolution reaction (OER) occurring at the anode. Many efforts have been made to improve the kinetics through careful design of highly active OER catalysts, especially with 3d transition metal oxides.^3,4^ Of these, Ni/Fe-based layered double hydroxide (LDH) materials have emerged as the most promising OER catalysts.^5,6^ To enhance catalytic efficiency, efforts have led to the development of materials where their surface undergoes *in situ* formation of an amorphous metal oxide/(oxy)hydroxide, while the core of the material remains unchanged.^7^ Since the as-synthesized materials themselves might not participate in the catalysis, these materials are often described as precatalysts and often contain other nonmetal dopants. Among them, transition-metal phosphides (TMPs) have emerged as the most promising candidate due to their improved corrosion resistance and exceptional electrical conductivity.^8,9^ Under applied anodic oxidation potentials, the surfaces of these materials undergo *in situ* transformation to (oxy)hydroxides, forming the active phase at the surface with their OER activity comparable or even superior to the leading Ni/Fe LDH catalysts.

To improve the OER activity of TMPs, bimetallic phosphides were developed to alter the electronic environment.^7−10^ The activities of these bimetallic phosphides follow the same trends observed for bimetallic hydroxide films, revealing substantially improved activity for Fe-doped NiP_*x*_ nanomaterials and composites.^8,9,11^ Furthermore, these Fe-doped TMPs have higher OER activity over the Ni/Fe (oxy)hydroxide counterparts due to the tuning of the Ni/Fe electronic structure by phosphorus. This change of the electronic structure results in an increase in the average oxidation state of Ni and Fe, which increases conductivity and accelerates the charge transport process.^8,9,11^ Although the activity trend of bimetallic phosphide and hydroxide films is similar, hydroxide undergoes complete oxidation, while only the surface of phosphides participates in the OER. For phosphides, additional factors can be applied to further improve their activity, such as their tunable electrical conductivity based on their composition, and the formation of the active phase depends upon their crystalline phase. For example, in NiFe alloys, amorphous structures were reported to offer more catalytic active sites for OER.^12,13^ The lack of Ni short-range order in amorphous TMPs contributes to the formation of coordinatively unsaturated Ni–Ni bonding. These metal sites with poor short-range coordination more easily participate in the *in situ* reconstruction to the amorphous Ni/Fe hydroxide phase at the surface compared to crystalline counterparts.^14^ Furthermore, the activity of bimetallic TMPs can be controlled by altering the elemental distribution in the material. Specifically, a core–shell morphology allows the material to retain some monometallic properties of each metal, which can give rise to synergistic properties, ultimately improving catalytic performance.^10,15−18^ Despite a growing number of studies on the precatalysts,^19^ new design of nanostructures with controllable reconstruction chemistry is needed for desired active catalysts in applications.

In this work, we synthesize NiP_*x*_-FeO_*x*_ core–shell structures for enhanced OER and investigate the structural evolution of the core–shell nanocatalyst before and after electrochemical treatment. Complementary characterization techniques are employed to reveal a thorough picture of the structural changes of the core–shell structure during cyclic voltammetry (CV) cycling. The electrochemical activities of the materials are correlated with the morphological/composition changes uncovered by transmission electron microscopy (TEM), and the changes of metal coordination environments are unveiled by X-ray absorption spectroscopy (XAS). Moreover, the structural evolution of the core–shell structures is followed by *ex situ* XAS after 1, 5, 10, 20, and 30 CV cycles. The data of the X-ray absorption near-edge structure (XANES) and the extended X-ray absorption fine structure (EXAFS) are analyzed and compared with those of monometallic NiP_*x*_ and FeO_*x*_ nanoparticles. Based on the results, we verified the chemical reactions involving the oxidation of Ni phosphides and observed the formation of the hydroxide surface during CV cycling. We further elucidated the importance of the Fe incorporation and the importance of core–shell morphology for improved OER activity.

## Experimental Methods

### Chemicals and Materials

Nickel(II) 2,4-pentadionate (Ni(acac)_2_, 95%), iron(III) 2,4-pentadionate (Fe(acac)_3_), tri-*n*-octylphosphine (TOP, 90%,), chloroform (CHCl_3,_ 99.8%+), and toluene were purchased from Alfa Aesar. Oleylamine (OLAM, 70%), 1-octadecene (ODE, 90%), methoxypolyethylene glycol acetic acid (PEG-COOH, M.W. = 5,000), and potassium hydroxide pellets (KOH, ACS) were obtained from Sigma-Aldrich. Iron pentacarbonyl (Fe(CO)_5_, 99.5%) was purchased from Acros Organics. Ethanol (200 proof) was obtained from Koptic. Hexane was purchased from EMD. Ultrapure H_2_O (18 MΩ) was used unless otherwise specified.

### Synthesis of NiP_*x*_ Nanoparticles

The NiP_*x*_ nanoparticles were synthesized by thermal decomposition of Ni(acac)_2_ in the presence of TOP. Typically, 103 mg of Ni(acac)_2_ (0.40 mmol), 8 mL of ODE, and 2 mL of OLAM were subsequently added to a 50 mL three-necked round-bottom flask equipped with a glass thermocouple, magnetic stir bar, and a condenser with water cooling. The reaction mixture was degassed for ∼10 min under Ar. After degassing, 2 mL of TOP was added to the reaction mixture followed by heating of the reaction mixture to 220 °C and keeping at 220 °C for 30 min. After the duration, the flask was allowed to cool to 50 °C, and then the product was collected by centrifuging in a mixture of 1:6 toluene/ethanol at 8000 relative centrifuge force (rcf) for 5 min. The NiP_*x*_ nanoparticles were suspended in ∼3 mL of toluene or another solvent as specified below for further use.

### Synthesis of NiP_*x*_-FeO_*x*_ Core–Shell Nanoparticles

The core–shell nanoparticles were synthesized by the thermal decomposition of Fe(CO)_5_ in the presence of the NiP_*x*_ nanoparticles. The NiP_*x*_ nanoparticles were suspended in a solution of 10 mL of ODE and 0.4 mL of OLAM via sonication. The reaction mixture was transferred to a 25 mL three-necked round-bottom flask, allowed to degas for 10 min, and heated to 110 °C. Once the temperature was stable at 110 °C, 20 μL of filtered Fe(CO)_5_ was injected into the reaction mixture and heated to 200 °C at a rate of 2.5 °C/min. After the reaction was allowed to proceed for 60 min, the product was purified by centrifuging with 1:6 toluene/ethanol mixture twice at 8000 rcf for 5 min. After purification, the core–shell nanoparticles were suspended in ∼3 mL of toluene.

### Synthesis of FeO_*x*_ Nanoparticles

The FeO_*x*_ nanoparticles were synthesized following the same procedure as the core–shell nanoparticles but in the absence of NiP_*x*_ nanoparticles. For this synthesis, 20 μL of filtered Fe(CO)_5_ was injected into a mixture containing 4 mL of ODE and 1 mL of OLAM in a 25 mL three-necked round-bottom flask.

### Ligand Exchange Process

The nanoparticles capped by organic ligands were transferred from the organic phase to the aqueous phase through the ligand exchange process with PEG-COOH. Briefly, each sample suspended in toluene was added to a 10 mL solution of 1 mg/mL PEG-COOH in chloroform. The mixture was stirred at 450 rpm for 8 h, followed by addition of 20 mL of hexane and centrifuging at 9000 rcf for 10 min. The pellet was dispersed in ∼10 mL of ethanol and recollected by centrifuging at 14-000 rcf for 30 min. The product was purified with water once, and ∼1.5 mL of ethanol was dispersed for further use.

### Characterization

Low-magnification TEM images were captured using a JEOL JEM-1011 microscope with an accelerating voltage of 100 kV. High-angle annular dark-field (HAADF)-scanning transmission electron microscopy (STEM) images were acquired using a JEOL ARM200F microscope equipped with a cold field emission gun and double aberration correctors operated at the accelerating voltage of 200 kV. The inner and outer collection angles for HAADF images used were 67 and 275 mrad, respectively. The convergent semiangle was 21 mrad. The spatial resolution of HAADF images was about 0.8 Å. The two-dimensional electron energy loss spectroscopy (EELS) mapping of the O K-edge, Fe L-edge, Ni L-edge and P K-edge was carried out using a Gatan GIF Continuum K3 System operated at an accelerating voltage of 200 kV with a collection semiangle of 90 mrad. Dispersion of 0.9 eV/channel was used to simultaneously acquire the Fe L-edge and Ni L-edge, as well as O K-edge and P K-edge. The dual-EELS mode was used for the energy-loss calibration. X-ray diffraction (XRD) was performed using a benchtop X-ray diffractometer (Rigaku Miniflex II) operated at 30 kV/15 mA with Cu Kα radiation as the source. X-ray photoelectron spectroscopy (XPS) was conducted at a base pressure of <5 × 10^–9^ Torr with a hemispherical electron energy analyzer (SPECS, PHOIBOS 100, MCD-5) and twin anode X-ray source (SPECS, XR50). The radiation source was Al Kα (1486.6 eV), which was used at 15 kV and 20 mA. The analyzer and X-ray source had an angle of 45° between them, and photoelectrons were collected along the sample surface normal. Each sample dispersed in ethanol was drop-casted onto a clean surface of 0.5 × 0.5 cm^2^ silicon wafer. The silicon wafer was then mounted to the sample plate, which was grounded via electrically conductive tape/wire for charge neutralization. The XPS spectra were calibrated using adventitious carbon located at 284.6 eV. CasaXPS was used to analyze the XPS spectra. The metal concentrations of each sample were obtained from an inductively couple plasma mass spectrometer (Thermo Fisher iCAP TQ ICP-MS).

### Electrochemical Treatments

An ink containing 5 mg/mL nanoparticles in a 0.1% Nafion/ethanol solution was prepared. The working electrode was prepared by drop-casting 200 μL of each ink solution on a carbon paper substrate over a 1 × 1 cm^2^ area for a total mass loading of 1 mg/cm^2^. CV was performed in purified Fe-free 1 M KOH^18,20^ using an SP-150 Biologic in a 3-electrode setup with a graphite carbon counter electrode and an Ag/AgCl (DryRef) reference electrode. All potentials were converted to be vs RHE using the equation *E*_RHE_ = *E*_Ag/AgCl_ + 0.059pH + *E*^o^_Ag/AgCl_ = *E*_Ag/AgCl_ + 1.023 V (*E*^o^_Ag/AgCl_ = 0.197 V vs NHE and pH = 14 at 1 M KOH). Upon completion of each treatment, the working electrode was quickly removed, thoroughly rinsed with water, and allowed to air-dry.

### X-ray Absorption Spectroscopy Data Collection and Analysis

XAS was performed at beamline 4-1 of the Stanford Synchrotron Radiation Lightsource (SSRL) at the SLAC National Accelerator Laboratory. Each Ni or Fe reference sample (∼60 μg/cm^2^, 0.5 cm-diameter circle) or electrochemically treated sample on a carbon paper substrate (cut into 1 × 0.5 cm^2^) was sandwiched between two pieces of Kapton tape. These reference samples and electrochemically treated samples were then placed in the beam path at a 45° angle from the incident X-ray beam. The fluorescence signal was collected using a Passivated Implanted Planar Silicon (PIPS) detector for the Fe K-edge (7112.0 eV) and Ni K-edge (8333.0 eV) at a 90° angle from the incident X-ray beam. Energy calibration was achieved by simultaneously scanning Fe and Ni foil references with each sample. Data calibration and analysis were carried out using the Demeter software package.^21^ EXAFS modeling was executed using Artemis from the Demeter software package.^21^ The absorption edge energies for Fe and Ni K-edges were calibrated to 7112.0 and 8333.0 eV, respectively, and the EXAFS models were optimized in the *R*-space using *k*^1^, *k*^2^, and *k*^3^ weightings obeying the Nyquist criterion. Based on various treatments, selective phases of α-Ni(OH)_2_,^22^ NiOOH,^23^ Ni_2_P,^24^ and Ni_3_(PO_4_)_2_^25^ were used to model Ni EXAFS spectra, while lepidocrocite (γ-FeOOH)^26^ and hematite (α-Fe_2_O_3_)^27^ were used to model Fe EXAFS data. The amplitude reduction factor (*S*_0_^2^) was determined by modeling the EXAFS spectra of pure metallic Ni and Fe reference foils, respectively. They have the same value of 0.76. The following Fourier transform (FT) parameters were chosen for the Ni K-edge; *k*_min_ = 3.0 Å^–1^, *k*_max_ = 12 Å^–1^, d*k* = 1, *r*_min_ = 1 Å, *r*_max_ = 3.5 Å, and dr = 0 with *k-*spline at 12 Å^–1^. For the Fe K-edge, the following FT parameters were adjusted to be *k*_max_ = 13 Å^–1^, with a *k*-spline at 13 Å. The resulting Δ*E*_0_, coordination number (CN), *R*, and σ^2^ factor are evaluated using Artemis FEFF simulation software.^21^ Δ*E*_0_ was constrained to be the same for all paths in a single fit. The σ^2^ factors for M–O paths from oxide/hydroxide/oxyhydroxide in each fit were set to be the same. The σ^2^ factors for the M–M and M–P paths in each fit were set to be the same. The CN and *R* are guessed independently.

## Results and Discussion

**Figure 1:**
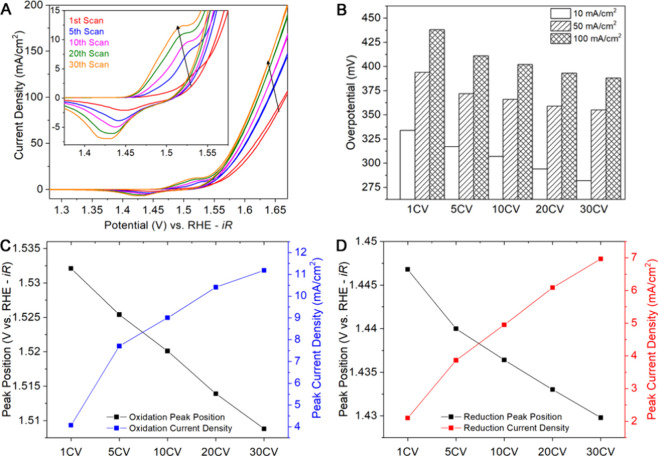
(A) CV profiles of the core–shell nanoparticles after 1, 5, 10, 20, and 30 CV cycles from 1.0 to 1.8 V vs RHE in 1 M KOH at a scan rate of 10 mV/s with the inset of zoom-in Ni^2+^/Ni^3+/4+^ redox region, (B) OER overpotentials as a function of cycles at current densities of 10, 50, and 100 mA/cm^2^, and (C, D) peak position and current density of the Ni^2^ → Ni^3+/4+^ oxidation region (C) and the Ni^3+/4+^ → Ni^2+^ reduction region (D) as a function of cycles.

The CV cycles of the core–shell nanoparticles were performed at a scan rate of 10 mV/s from 1.0 to 1.8 V vs RHE for a total of 30 cycles. As the number of cycles increases, the overpotential decreases, indicating an increase in the OER activity (Figure 1A). Quantitative analysis shows that the overpotential decreases faster from the first cycle to the fifth cycle than the next five cycles, gradually levels off, and becomes stable at the 30th cycle (Figure 1B). The overpotential values decrease from 334 mV at 10 mV/cm^2^ and 438 mV at 100 mV/cm^2^ at the first CV cycle to 228 mV at 10 mV/cm^2^ and 388 mV at 100 mV/cm^2^ at the 30th cycle. In addition to the OER activity, the corresponding changes in the Ni^2+^/Ni^3+/4+^ redox region were observed where the redox peak shifted to a lower potential and the redox current increased as the number of cycles increased. The Ni^2+^/Ni^3+/4+^ oxidative region was overlapped with the OER onset potentials. To account for the current density increase due to the OER, we fitted the baseline using the polynomial function and subtracted it from the Ni oxidative region to retrieve the baseline-corrected peak that was solo-contributed from Ni^2+^/Ni^3+/4+^ oxidation (Figure S1). The potential shifts and the integration of the redox currents were plotted as a function of increased number of cycles for both the regions of oxidation (Figure 1C) and reduction (Figure 1D). A similar trend was observed with a decrease in peak potential by ∼20 mV and an increase in peak current density of ∼6 mA/cm^2^ from 1 to 30 CV cycles. The Ni^2+^/Ni^3+/4+^ redox potential shifts to lower values, while the OER overpotential decreases, suggesting that an increase in Ni incorporation to the Fe shell facilitated the OER. These changes became smaller and the CV profiles eventually reached convergence toward 30 cycles with little change after 25 CV cycles (Figure S2). Compared to the core–shell nanoparticles, the NiP_*x*_ nanoparticles were much less active for OER with the overpotential of 400 mV at the current density of 10 mV/cm^2^, while the FeO_*x*_ nanoparticles were inactive for OER (Figure S3).

**Figure 2:**
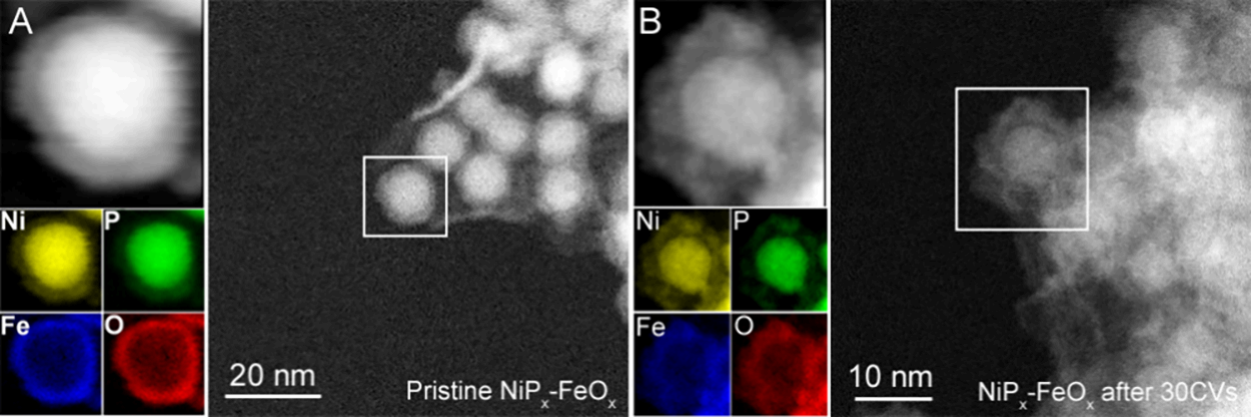
TEM characterization of the core–shell nanoparticles before (A) and after (B) 30 CV cycles from 1.0 to 1.8 V vs RHE in 1 M KOH at a scan rate of 10 mV/cm^2^. HAADF-STEM images show the core–shell morphology, and the EELS maps indicate the distribution of elements Ni (yellow), P (green), Fe (blue), and O (red).

Structural characterization of the core–shell nanoparticles was carried out before and after the 30 CV cycles from 1.0 to 1.8 V vs RHE at a scan rate of 10 mV/cm^2^ by TEM and XPS. Figure 2 shows the TEM characterization of the core–shell nanoparticles before and after 30 CV cycles. The as-synthesized core–shell nanoparticles consist of a NiP_*x*_ core and an FeO_*x*_ shell, where some Ni and P are present due to the diffusion at the core–shell interfaces. After 30 CV cycles, the core–shell morphology remains, but the core becomes slightly smaller, while the shell grows thicker and more porous. Histograms of the size distribution obtained from the low-magnification TEM images also support the STEM/HAADF analysis (Figure S4). At the same time, there appears to be a gap rising between the core and the shell that can be attributed to the Kirkendall effect of the interdiffusion between Ni/P and Fe. The increases of Ni and the porosity in the shell account for the change of the Ni^2+^/Ni^3+/4+^ redox region (i.e., potential shift to the lower value and the increase in current). The quantitative XPS analysis listed in Table S1 indicates that the Ni:Fe ratio increases from 1.14 to 1.22. Since XPS is surface-sensitive, the increase in Ni/Fe ratio supports the TEM-EELS observation of the Ni migration from the core to the shell within the nanoparticle after cycling. Meanwhile, the content of P decreases after 30 CV cycles as indicated by the increase in the ratio of Ni to P from 1.92 to 3.73.

**Figure 3:**
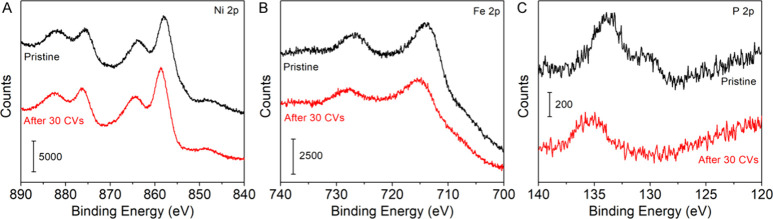
XPS spectra of the core–shell nanoparticles before (in black) and after (in red) 30 CV cycles from 1.0 to 1.8 V vs RHE in 1 M KOH at a scan rate of 10 mV/cm^2^: (A) Ni 2p, (B) Fe 2p, and (C) P 2p.

Further analysis of the XPS spectra obtained from the core–shell nanoparticles before and after 30 CV cycles reveals the oxidation state changes after the electrochemical treatments. The Ni 2p_3/2_ XPS spectrum exhibits a major peak at 857.6 eV with satellite splitting of ∼6 eV that can be assigned to oxidized Ni (e.g., Ni(OH)_2_ or Ni_3_(PO_4_)_2_)^28^ and a shoulder peak at 854.2 eV that corresponds to the electron-rich Ni in NiP_*x*_ (Figure 3A). After 30 CV cycles, the major peak slightly shifts to a higher binding energy of 858.4 eV, while the shoulder peak disappears, indicating a more electron-deficient oxidized Ni. Similarly, Fe became more oxidized as indicated by the Fe 2p_3/2_ binding energy shifted from 713.6 to 714.8 eV after 30 CV cycles (Figure 3B). Accompanied by the oxidation of Ni and Fe, P was also oxidized from phosphide to phosphate after 30 CV cycles. The P 2p XPS spectrum of the pristine sample exhibits higher binding energies at 133.8 eV that can be assigned to oxidized P (e.g., PO_4_^3–^, 134.0 eV) and 130.2 eV that can be attributed to P from NiP_*x*_ (negatively by 0.2 eV from red phosphorus P 2p of 130.4 eV).^28^ After 30 CV cycles, only one major peak was observed at 135.1 eV, suggesting that the surface was dominated by phosphate and was electron-deficient.

**Figure 4:**
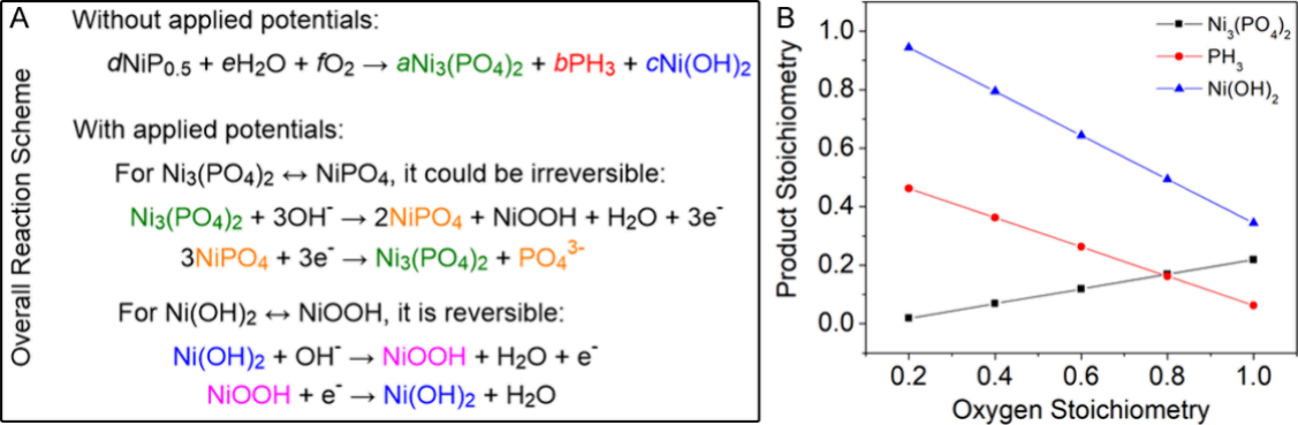
(A) Plausible chemical reaction schemes of NiP_*x*_ with and without applied potentials. (B) Plot of the stoichiometric coefficients of the products Ni_3_(PO_4_)_2_, PH_3_, and Ni(OH)_2_ as a function of the oxygen stoichiometry.

We deduce plausible chemical reactions of NiP_*x*_ with and without applied potentials. Figure 4A illustrates the overall reaction schemes. Without applied potentials, NiP_*x*_ would convert to Ni_3_(PO_4_)_2_, PH_3_, and Ni(OH)_2_ during ligand exchange due to the presence of H_2_O and air. The hypothesis is supported by the XPS data in Figure 3 showing evidence of Ni_3_(PO_4_)_2_ and Ni(OH)_2_. Gas is formed during the process, which we hypothesize to be PH_3_ due to the limited amount of available O_2_ under the reaction condition. To further verify the hypothesis, we immerged the pristine sample into 1 M KOH for 12 h. The XPS results in Figure S5 show that most of NiP_*x*_ was converted to Ni_3_(PO_4_)_2_ and Ni(OH)_2_.

Based on XPS and EELS analysis, the Ni/P ratio is approximately 2:1 with a stoichiometry of Ni_2_P. In this case, *x* in NiP_*x*_ can be set as 0.5. We then balance the chemical reaction of *d*NiP_0.5_ + *e*H_2_O + *f*O_2_ → *a*Ni_3_(PO_4_)_2_ + *b*PH_3_ + *c*Ni(OH)_2_ based on mass conservation. Four linear equations involving Ni, P, H, and O can be solved to obtain stoichiometric coefficients: *a*, *b*, *c*, *d*, *e*, and *f*. The detailed derivation for the solution is provided in the Supporting Information. It is found that the stoichiometric ratio of the product changes as a function of the abundance of oxygen (*f*), as shown in Figure 4B. The product is dominated by Ni(OH)_2_ when oxygen is deficient. At the oxygen level *f* = 0.2, only a small amount of Ni_3_(PO_4_)_2_ is formed and the formation of gaseous PH_3_ is the main pathway for P elimination. As the oxygen stoichiometry increases, the amount of Ni_3_(PO_4_)_2_ increases and that of Ni(OH)_2_ decreases. The PH_3_ pathway becomes less important. At the oxygen level of *f* = 1, the amounts of Ni_3_(PO_4_)_2_ and Ni(OH)_2_ produced are comparable and the loss of P due to PH_3_ formation is greatly minimized.

The difference between the oxygen-deficient (*f* = 0.2) and oxygen-rich (*f* = 1) scenarios resembles the variance between ligand exchange in air and under an oxygen-saturated environment similar to those under the OER condition. Under applied voltages, the products of the NiP_*x*_ oxidation, Ni_3_(PO_4_)_2_ and Ni(OH)_2_, are expected to undergo the electrochemical reactions shown in Figure 4A. During oxidation half-reaction, Ni_3_(PO_4_)_2_ would be oxidized to NiPO_4_ and NiOOH under alkaline condition (Ni_3_(PO_4_)_2_ + 3OH^–^ → 2NiPO_4_ + NiOOH + H_2_O + 3e^–^). At the reduction half-reaction, NiPO_4_ would be reduced to Ni_3_(PO_4_)_2_ and lose a PO_4_^3–^ as an electrolyte (3NiPO_4_ + 3e^–^ → Ni_3_(PO_4_)_2_ + PO_4_^3–^). As for Ni(OH)_2_ and NiOOH, the redox chemistry follows Ni(OH)_2_ + OH^–^ ↔ NiOOH + H_2_O + e^–^.

**Figure 5:**
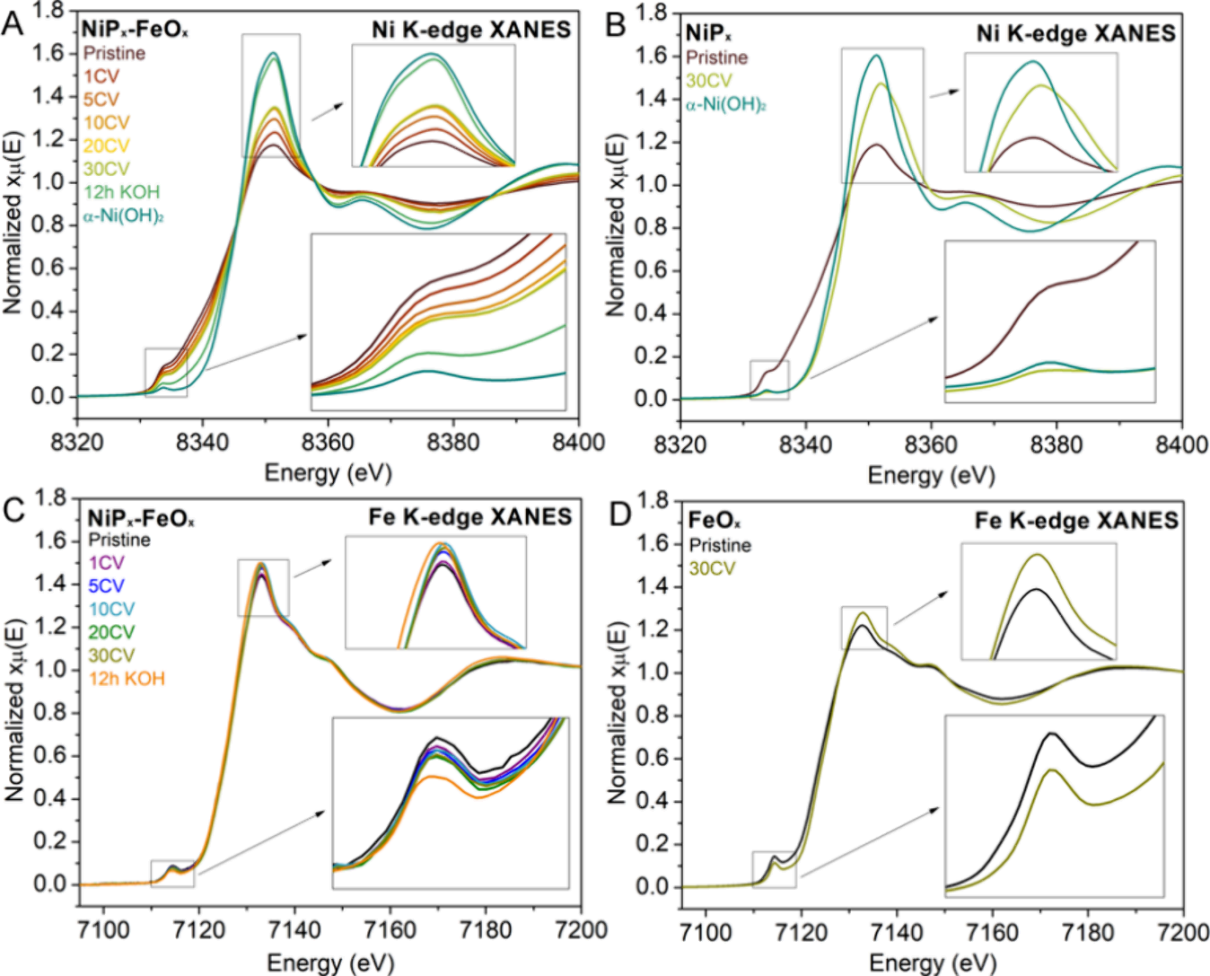
(A, B) Ni K-edge XANES spectra of the NiP_*x*_-FeO_*x*_ core–shell nanoparticles (A) and NiP_*x*_ (B) with different treated conditions. The spectrum of α-Ni(OH)_2_ is included as a comparison. (C, D) Fe K-edge XANES spectra of the NiP_*x*_-FeO_*x*_ core–shell nanoparticles (C) and NiP_*x*_ (D) with different treated conditions.

To further elucidate the structural evolution due to electrochemical cycling, we performed XAS on the NiP_*x*_-FeO_*x*_ core–shell nanoparticles before and after cycling and compared the data to that from monometallic NiP_*x*_ and FeO_*x*_ nanoparticles. Given the above reaction schemes, we analyzed the XAS spectra and revealed the oxidation state and coordination environment changes of the nanostructures. The Ni K-edge and Fe K-edge XANES spectra of the NiP_*x*_-FeO_*x*_ core–shell nanoparticles along with NiP_*x*_ and FeO_*x*_ nanoparticles are plotted in Figure 5. Differences in the pre-edge and edge intensities arise from changes in the coordination environment and oxidation state of the absorbing atom. For Ni and Fe, the intense edge absorption peak comes from the dipole-allowed 1s → 4p transition, while the weak pre-edge feature is associated with the dipole-forbidden, quadrupole-allowed 1s → 3d transition.^29−32^ The 1s → 4p transition energy is directly related to the effective charge of the absorbing atom,^33,34^ and therefore, a higher intensity would suggest that more oxygen atoms are coordinated with the absorbing atom.^29,34^ The peak position of the absorption edge energy is an indicator of the oxidation state, where a lower edge energy value is associated with a higher oxidation state. The 1s → 3d transition is dipole-forbidden but quadrupole-allowed along with vibronic coupling, which yields weak pre-edge features.^35^ Pre-edge features become more intense in the noncentrosymmetric system due to 1s transitions to the p component of a 4p-3d hybridized orbital, which was often found in NiFe OER catalysts.^36,37^

For the Ni K-edge, compared to the pristine core–shell sample without treatment, a significant dampening of the pre-edge feature and an increase in edge intensity were observed upon CV cycling (Figure 5A). The changes appeared after the first CV cycle and reached a plateau toward the 30th cycle. This suggests that Ni adopts a distorted octahedral geometry and that the oxygen coordination increases as the Ni geometry becomes more ordered. For comparison, the Ni K-edge XANES of the core–shell nanoparticles immerged in the 1 M KOH electrolyte without applied potentials appeared similar to that of α-Ni(OH)_2_ with almost the same edge intensity. Without the shell, NiP_*x*_ nanoparticles exhibited a noticeable difference in the structure obtained after 30 CV cycles (Figure 5B). Compared to the dry NiP_*x*_ sample, not only the CV-treated sample has a drastic increase in the edge intensity but also the peak shifted to a lower absorption edge energy. This shift could be attributed to the existence of NiOOH,^38,39^ which was often associated with the conversion of the active, highly unstable γ-NiOOH to the more stable, less active β-NiOOH mixed with β-Ni(OH)_2_.^40−43^ These results suggest that the FeO_*x*_ shell helps stabilize α-Ni(OH)_2_ and γ-NiOOH phases, which are the most active phases for OER based on the previously reported NiFe catalysts.^39,40,44,45^ In fact, the STEM/EELS characterization in Figure 2 indicates that the shell consists of Ni-incorporated FeO_*x*_/Fe(OH)_*x*_.

As for Fe, the electrochemical CV cycles do not have a significant effect on the coordination environment and oxidation state of Fe in the shell of the core–shell structure by comparing the Fe K-edge XANES spectra (Figure 5C). There was a slight increase in the edge intensity and a small decrease in the pre-edge intensity after each treatment; however, the changes were much less significant than those of the Ni K-edge. As a comparison, the FeO_*x*_ nanoparticles exhibited relatively higher oxygen coordination and less distortion after 30 CV cycles as indicated by the changes observed in the Fe K-edge XANES before and after 30 CV cycles (Figure 5D). As it can be seen, the Fe K pre-edge is featured with a single, broad band for Fe in both the Ni-incorporated FeO_*x*_/Fe(OH)_*x*_ shell and pure FeO_*x*_ nanoparticles, suggesting that Fe adopts an octahedral geometry.^46^ Compared with the pure FeO_*x*_ nanoparticles, Ni incorporation to the FeO_*x*_/Fe(OH)_*x*_ shell leads to a broader and less intense pre-edge feature.

**Figure 6:**
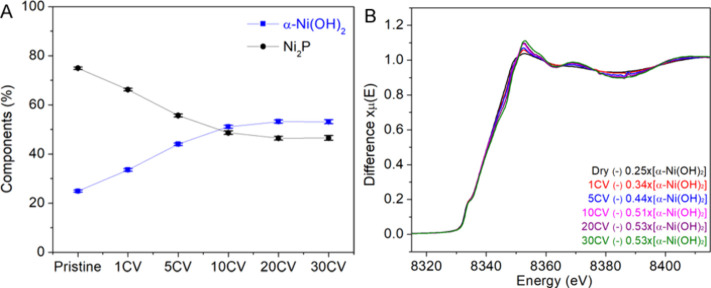
(A) Plot of percent distribution of Ni_2_P and α-Ni(OH)_2_ in the core–shell catalyst determined by the LCF analysis. (B) Subtracted spectra of pristine (dry) and 1, 5, 10, 20, and 30 CV-treated NiP_*x*_-FeO_*x*_ with subtraction weights of α-Ni(OH)_2_ for the Ni K-edge XAS.

To look into the conversion of NiP_*x*_ → α-Ni(OH)_2_ of the core–shell nanoparticles, we further analyzed the Ni K-edge XANES spectra by linear combination fitting (LCF) using XAS data of Ni_2_P nanoparticles^47^ and α-Ni(OH)_2_. The results are shown in Figure 6A. For the pristine (as-synthesized) sample, it was composed of a mixture of 75% NiP_*x*_ and 25% α-Ni(OH)_2_. The conversion of NiP_*x*_ to α-Ni(OH)_2_ was the result of the ligand exchange process where NiP_*x*_ was in contact with water and air. As the number of cycles increased, the content of α-Ni(OH)_2_ increased from 34% for cycle 1 to 53% for cycle 20 and plateaued at cycle 30. From the residual plots of the LCF, variations between the fits and experimental data increased with the number of cycles, suggesting that an additional component could be present (Figure S6). Given that Ni_3_(PO_4_)_2_ was a plausible product from the NiP_*x*_ → α-Ni(OH)_2_ conversion in the presence of oxygen, we plotted the subtracted spectra of each untreated spectrum minus the corresponding contribution from the α-Ni(OH)_2_ spectrum (Figure 6B). The subtracted spectra show an increase in the edge intensity with an increased number of cycles, indicating that the contribution of Ni_3_(PO_4_)_2_ becomes more significant.

**Figure 7:**
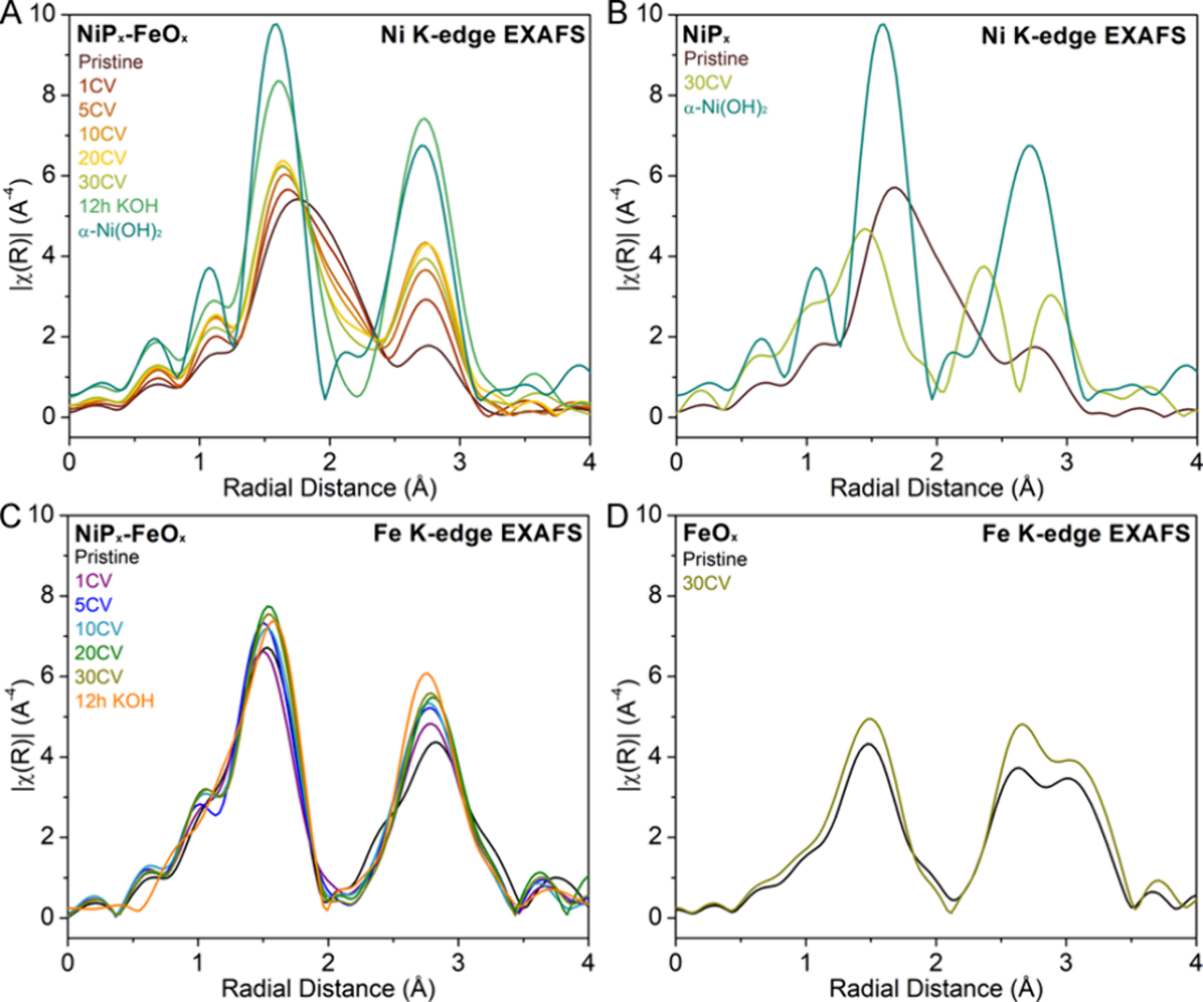
(A, B) Ni K-edge *k*^3^-weighted FT EXAFS spectra for the pristine and 1, 5, 10, 20, and 30 CV cycle-treated NiP_*x*_-FeO_*x*_ core–shell (A) and the pristine and 30 CV cycle-treated monometallic NiP_*x*_ nanoparticles (B). The spectrum of the α-Ni(OH)_2_ standard was included for comparison. (C, D) Fe K-edge *k*^3^-weighted FT EXAFS spectra for the pristine and 1, 5, 10, 20, and 30 CV cycle-treated NiP_*x*_-FeO_*x*_ core–shell (C) and the pristine and 30 CV cycle-treated monometallic FeO_*x*_ nanoparticles (D).

Analysis of the Ni and Fe K-edge EXAFS can reveal changes in coordination number and bond length of the core–shell nanoparticles before and after electrochemical processes. Due to the complexity of the structure, we first qualitatively analyze the Fourier transforms of the *k*^3^-weighted Ni and Fe K-edge EXAFS of NiP_*x*_-FeO_*x*_ core–shell nanoparticles before and after CV cycling. For the Ni K-edge, the first shell of the pristine NiP_*x*_-FeO_*x*_, typically associated with Ni–O scattering, is considerably broader than that of α-Ni(OH)_2_ (Figure 7A). The Ni coordination environment can be seen to undergo considerable changes in first-shell coordination after electrochemical cycling. The changes can be attributed to the contribution of multiple bonding distances in the first shell due to the chemical transformation from Ni_2_P to α-Ni(OH)_2_ and Ni_3_(PO_4_)_2_. The chemical transformation can also be observed in the core–shell nanoparticles after immersion in 1 M KOH for 12 h without applied voltages. Both the first and second shells of the KOH-treated sample closely resemble the α-Ni(OH)_2_ spectra, suggesting that the major product is α-Ni(OH)_2_. This observation agrees with the chemical reaction of Ni_2_P to α-Ni(OH)_2_ and Ni_3_(PO_4_)_2_ occurring at a low oxygen stoichiometry (e.g., *f* = 0.2) as shown in Figure 4, due to limited O_2_ solubility and diffusivity in a KOH solution compared to water.^48^ A similar change was observed for NiP_*x*_ nanoparticles by comparing Ni K-edge EXAFS before and after 30 CV cycling in Figure 7B; however, the radial distance after CV cycling is slightly shorter than that of α-Ni(OH)_2_ possibly due to the contribution from aged β-Ni(OH)_2_. This result suggests that the FeO_*x*_ shell or Fe incorporation improves the stability of the OER-active α-Ni(OH)_2_ by preventing it from aging to β-Ni(OH)_2_. In contrast to the Ni K-edge, the differences in the first and second shells of the Fe K-edge before and after treatment are subtle for both the NiP_*x*_-FeO_*x*_ core–shell and FeO_*x*_ nanoparticles as shown in Figure 7C,D, respectively. Nonetheless, the Ni core or incorporation of Ni into FeO_*x*_ appears to make a substantial difference in the second shell of Fe–M_*x*_ path contributions.

Quantitative analysis of the EXAFS data was performed through modeling based on the chemistry involved under the treated conditions. For the Ni K-edge, paths from the following crystal structures α-Ni(OH)_2_^22^ (Ni–O and Ni–Ni), Ni_2_P^24^ (Ni–P and Ni–Ni), Ni_3_(PO_4_)_2_^25^ (Ni–P), and NiOOH^23^ (Ni–O and Ni–Ni) are considered in the modeling. In the case of the Fe K-edge, paths from α-Fe_2_O_3_ (hematite)^27^ (Fe–O and Fe–Fe) and Fe γ-FeOOH (lepidocrocite)^26^ (Fe–O and Fe–Fe) are considered in the modeling. Best fit results from EXAFS modeling are listed in Tables S4 and S5. Fourier transforms of *k*^3^*-*weighted Ni and Fe K-edge EXAFS and their best fits of the real and imaginary components are displayed in Figures S7–S9. The Ni and Fe *k*^3^-weighted FT EXAFS and fits are shown in Figures S10 and S11.

**Figure 8:**
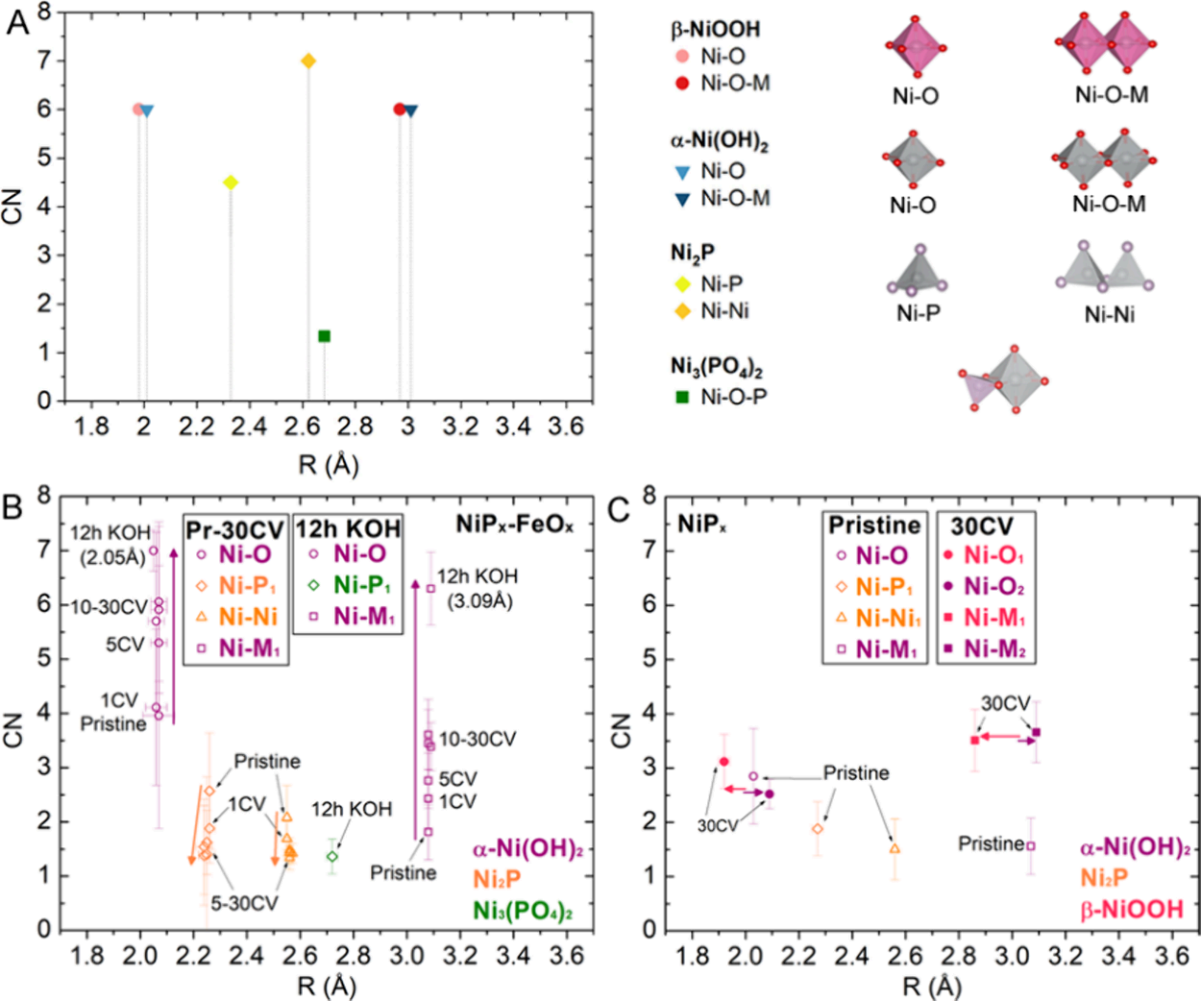
(A) Coordination number (CN) vs bond length (*R*) of the Ni paths used for the EXAFS modeling with their legends and geometries on the right (note that Ni_2_P also contains trigonal bipyramids); (B) CN vs *R* for the pristine (or Pr), 1, 5, 10, 20, and 30 CV cycle-treated, and KOH-treated NiP_*x*_-FeO_*x*_ core–shell nanoparticles; (C) CN vs *R* for pristine and 30 CV cycle-treated monometallic NiP_*x*_ nanoparticles.

**Figure 9:**
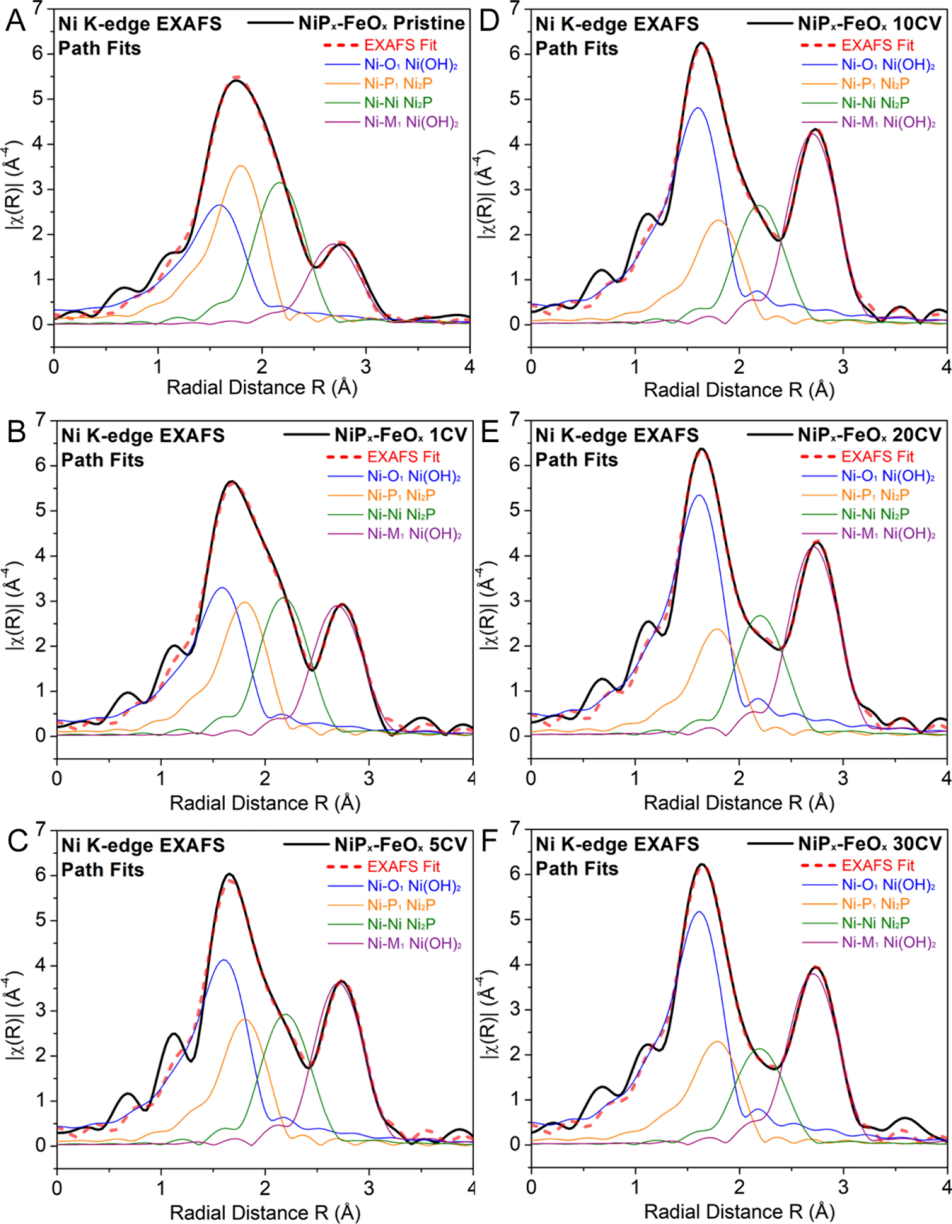
Ni path contributions of the Ni K-edge FT EXAFS fit for the (A) pristine and (B) 1, (C) 5, (D) 10, (E) 20, and (F) 30 CV cycle-treated NiP_*x*_-FeO_*x*_ core–shell nanocatalysts.

For the Ni K-edge, the geometric configurations of different paths used for the fits with their corresponding coordination numbers (CNs) and bond lengths (*R*) are illustrated in Figure 8A. The shortest bond at ∼2.0–2.1 Å corresponds to the Ni–O scattering paths arising from NiO_6_ octahedra that appear in both α-Ni(OH)_2_ and Ni_3_(PO_4_)_2_, while the longest length at ∼3.10 Å is associated with Ni–M paths from edge-sharing NiO_6_ octahedra, which are present in α-Ni(OH)_2_. The Ni–P bond length at ∼2.2–2.3 Å originates from NiP_4_ tetrahedra, which are bound together by shared vertices, giving rise to the Ni–Ni distance of ∼2.5–2.7 Å.^49−51^ The paths used for the Ni K-edge fitting depend on the composition of the nanoparticles and the chemistry proposed in Figure 4. The fit results are listed in Table S4 and plotted in Figure 8B,C to better visualize the changes of CN and *R* for individual paths. The individual path contributions of the Ni *k*^3^*-*weighted FT EXAFS fits of the core–shell nanostructures treated under different conditions are shown in Figure 9.

For the core–shell nanoparticles before and after CV, Fe incorporation prevents them from aging and the time immerged in the KOH is relatively short (less than 1.5 h), and therefore, they are mainly composed of NiP_*x*_ and α-Ni(OH)_2_. The Ni–P and Ni–Ni paths from Ni_2_P, as well as the Ni–O and Ni–M paths from α-Ni(OH)_2_, were used to model their Ni K-edge EXAFS. As can be seen in Figure 8C and Table S4, a significant decrease in coordination of the Ni–P and Ni–Ni paths is observed, while the Ni–O and Ni–M paths exhibit a simultaneous increase in coordination as the CV progresses. For the Ni–O and Ni–M bond distances originating from α-Ni(OH)_2_, a CN_Ni–O_:CN_Ni–M_ ratio of 1 is expected as both should have a CN of 6.^29,39,52,53^ However, the CN of the Ni–M path was observed to approach a lower value of 3.6 after 30 CV cycles due to the presence of the remaining NiP_*x*_ in the core. In contrast, the 12 h KOH-treated core–shell nanoparticles were best modeled using Ni–O and Ni–M paths (Figure S12A) from α-Ni(OH)_2_ with a single Ni–P path from Ni_3_(PO_4_)_2_ accounting for the remaining phosphate coordination from edge-sharing octahedral NiO_6_ and PO_4_^3–^ tetrahedra.^9,54^ Despite the similarity of the 12 h KOH-treated core–shell FT EXAFS to that of α-Ni(OH)_2_ (Figure S12B,C), the Ni–P path at 2.72 Å from Ni_3_(PO_4_)_2_ is needed to correctly model the KOH-treated core–shell catalyst, which validates the presence of Ni_3_(PO_4_)_2_.

**Figure 10:**
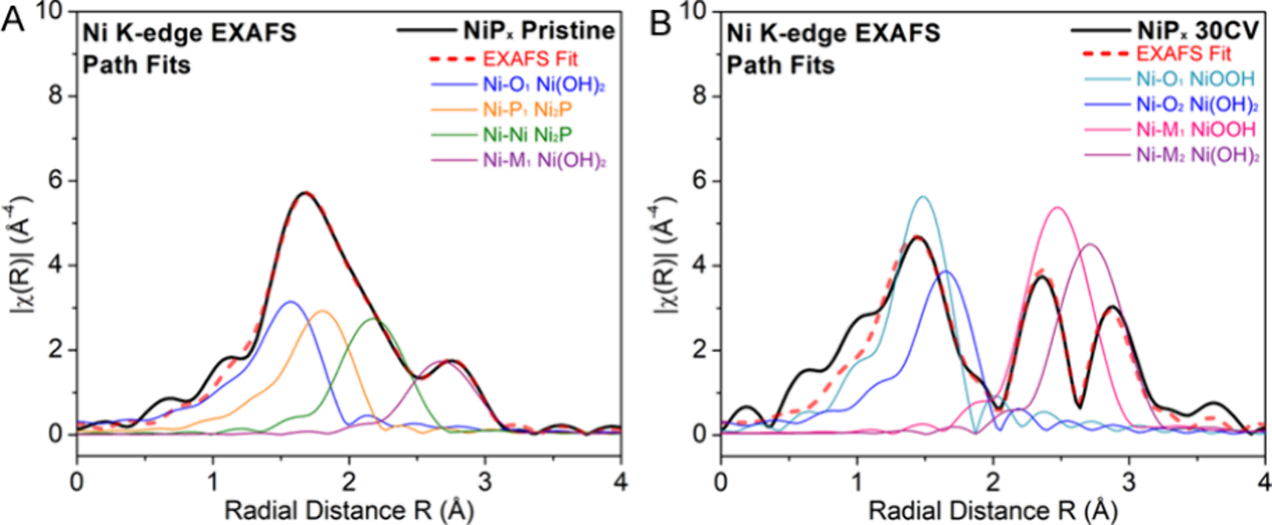
Ni path contributions of the Ni K-edge FT EXAFS fits for NiP_*x*_ nanoparticles before (A) and after 30 CV cycles (B).

As a comparison, the pristine NiP_*x*_ was modeled using the same paths as those for the core–shell; however, after 30 CV cycles, the NiP_*x*_ sample was better modeled using the Ni–O and Ni–M from Ni(OH)_2_ and NiOOH due to the faster conversion and the irreversible aging process (Figure 10). The fitting results in Figure 8D and Table S4 show that the pristine NiP_*x*_ has similar CN and *R* values to the pristine NiP_*x*_-FeO_*x*_ core–shell catalyst. However, after 30 CV cycles, the NiP_*x*_ nanoparticles undergo a complete loss of the Ni–P characteristics that can only be modeled using scattering paths from α-Ni(OH)_2_ and β-NiOOH. The Ni–O and Ni–M_*x*_ paths at 1.92 and 2.86 Å are associated with constrained NiO_6_ octahedra from NiOOH,^39,53,55^ while the scattering paths at 2.08 and 3.09 Å are associated with NiO_6_ octahedra from Ni(OH)_2_. Unlike the core–shell nanostructure, the presence of the paths from NiOOH in the 30 CV cycle-treated NiP_*x*_ indicates the relaxation of γ-NiOOH to more stable, less active β-NiOOH mixed with α/β-Ni(OH)_2_ after repeated CV cycles.^40−43,56^ These results are in agreement with those from the XANES analysis. We can conclude that the FeO_*x*_ shell helps stabilize the reduction of γ-NiOOH back to α-Ni(OH)_2_ after overcharging for improved OER activity.

**Figure 11:**
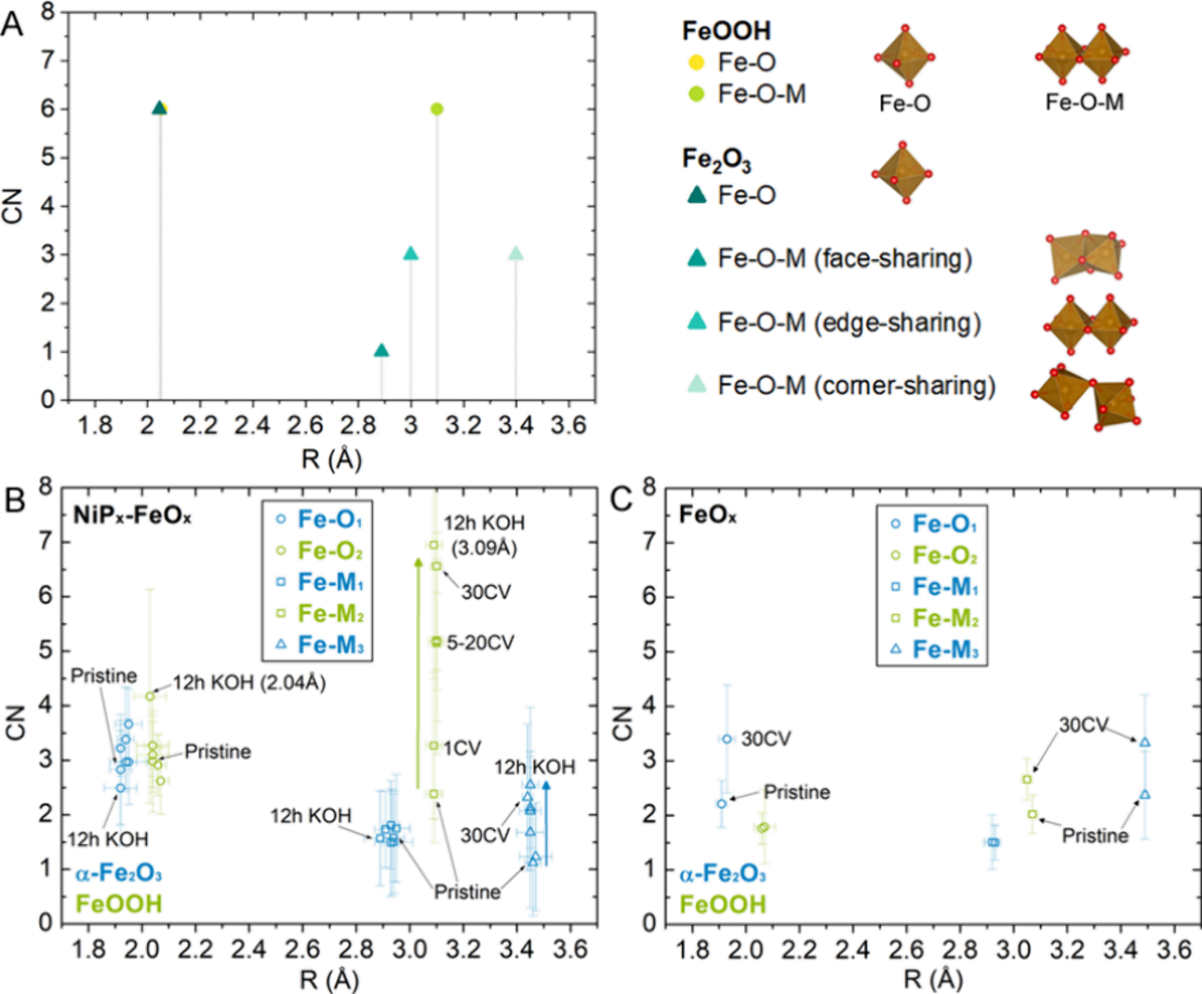
(A) Coordination number (CN) vs bond length (*R*) of the Fe paths used for the EXAFS modeling with their legends and geometries, (B) CN vs *R* for the pristine, 1, 5, 10, 20, and 30 CV cycle-treated, and KOH-treated core–shell nanoparticles, and (C) CN vs *R* for the pristine and 30 CV cycle-treated monometallic FeO_*x*_ nanoparticles.

**Figure 12:**
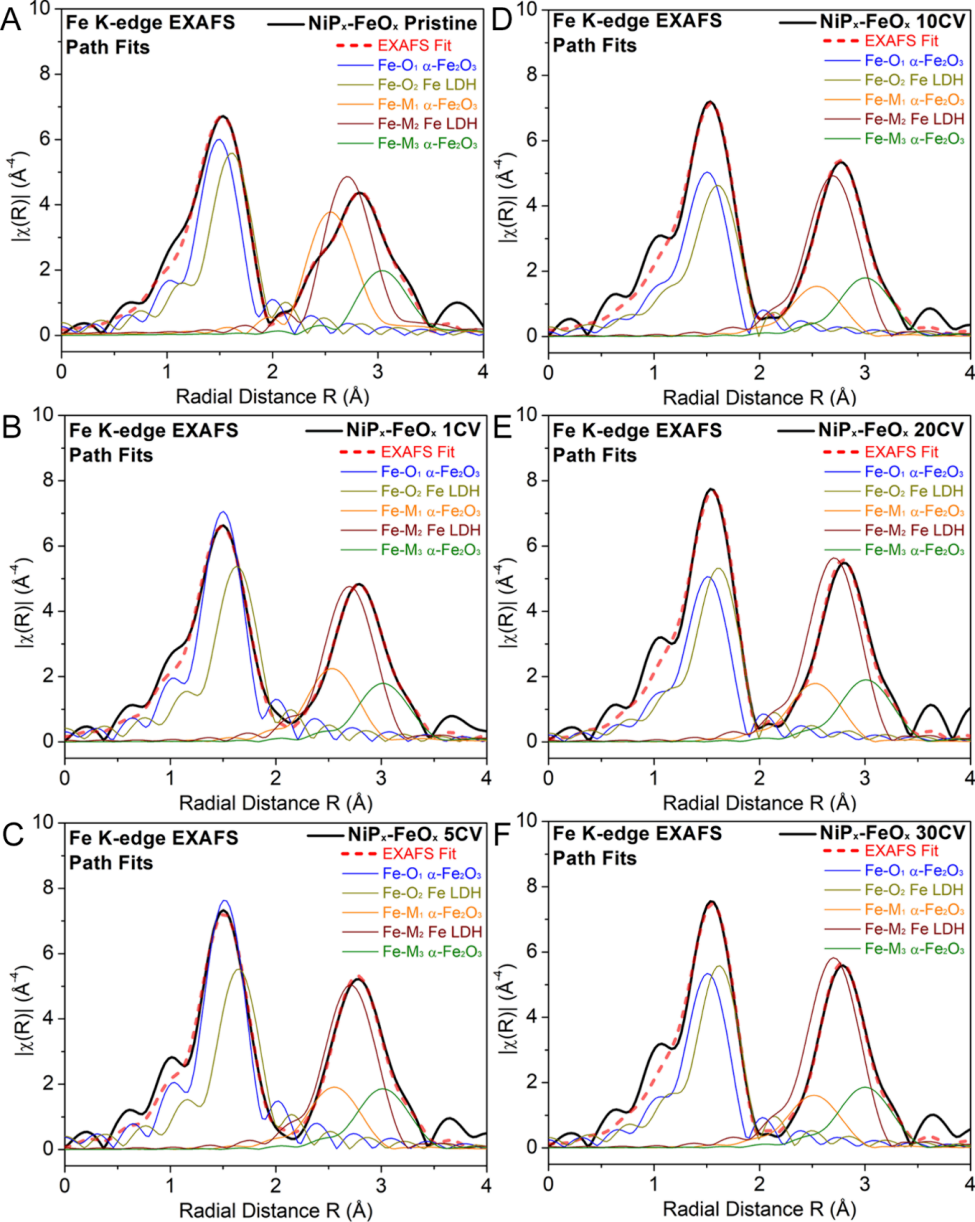
Fe path contributions of the Fe K-edge FT EXAFS fit for the (A) pristine and (B) 1, (C) 5, (D) 10, (E) 20, and (F) 30 CV cycle-treated NiP_*x*_-FeO_*x*_ core–shell nanocatalysts.

For the Fe K-edge, the FT EXAFS spectra are similar to those previously reported for hematite and FeOOH polymorphs such as α/β/γ-FeOOH, amorphous 2-line ferrihydrite, feroxyhite (δ-FeOOH), and other hydrated hematite-like (α-Fe_2_O_3_) structures.^46,57−62^ The first shell of these materials often contains a combination of Fe–O_*x*_ path contributions, which can often be modeled by simply fitting one or two Fe–O paths.^58,62^ Furthermore, these materials have three Fe–M_*x*_ path contributions in the second shell arising from Fe–M face-sharing, edge-sharing, and corner-sharing sites.^58−60,62^ Based on these previous studies, a 5-path model was applied for the fits of the Fe K-edge EXAFS spectra where Fe–O_1_, Fe–M_1_ (face/edge-sharing), and Fe–M_3_ (corner-sharing) paths were taken from the α-Fe_2_O_3_ crystal structure, and Fe–O_2_ and Fe–M_2_ (edge-sharing) paths were taken from the lepidocrocite (γ-FeOOH) crystal structure. The geometric configurations of different paths used for the fits with their corresponding CN values and *R* are illustrated in Figure 11A. The fit results are listed in Table S5 and plotted in Figure 11B,C to compare the changes of CN and *R* for individual paths. The individual path contributions of the Fe *k*^3^*-*weighted FT EXAFS fits for the core–shell nanostructures treated under different conditions are shown in Figure 12. As a comparison, the individual path contributions of the Fe *k*^3^*-*weighted FT EXAFS fits for the core–shell structures after 12 h immersion in 1 M KOH and for FeO_*x*_ before and after 30 CV cycles are shown in Figure S13.

To determine the Fe–O coordination, the average ⟨Fe–O⟩ bond length and total coordination number (total CN) of the Fe–O_*x*_ paths are both considered. The first shells of maghemite, magnetite, hematite, ferrihydrite, and the FeOOH polymorphs typically have two Fe–O bonds associated with the crystal structure.^58,59,62^ According to the literature, an average ⟨Fe–O⟩ bond length closer to 1.96 Å suggests the presence of tetrahedrally coordinated Fe (CN = 4), while an ⟨Fe–O⟩ bond length of ∼2.00 Å is expected for 100% octahedral Fe coordination (CN = 6).^62^ The average ⟨Fe–O⟩ bond distances and the total CN obtained from the fits are listed in Table S6. The ⟨Fe–O⟩ bond distances of the NiP_*x*_-FeO_*x*_ core–shell catalysts are close 1.99–2.00 Å with a total CN of ∼6, an indication of 100% octahedrally coordinated Fe. For comparison, without the NiP_*x*_ core, the pristine FeO_*x*_ nanoparticles has an ⟨Fe–O⟩ distance of 1.98 ± 0.11 Å and a CN of 4.0, suggesting that there might be tetrahedral sites present; however, after 30 CV cycle treatment, the total CN increases to 5.2.

For the second shell, the distance of the absorbing Fe atom to the closest adjacent metal neighbor depends on the symmetry, neighboring atom, and the linkage of structural units, which are referred to as face-, edge-, or corner-sharing sites. The distance of Fe–M with M referring to Fe_1_, Fe_2_, and Fe_3_ at the face-sharing, edge-sharing, and corner-sharing sites increases in the order of Fe–Fe_1_ at 2.89 Å, Fe–Fe_2_ at 2.97 Å, and Fe–Fe_3_ at 3.39 Å.^58−60,62,63^ Because the bond length is similar to the difference <0.08 Å, the reported value was the average of Fe–M distances of face-sharing/edge-sharing sites.^64^ For Ni/Fe layered hydroxides modeled using FeOOH polymorphs, the bond length at ∼3.0–3.2 Å is commonly associated with edge-sharing FeO_6_.^58−60,62^

In our case, the edge-sharing bond distance (Fe–M_2_) of the NiP_*x*_-FeO_*x*_ core–shell catalyst is at the length of 3.09–3.10 Å, but the CN of edge-sharing sites undergoes a significant increase, from 2.4 to 3.3, 5.2, 5.2, 5.1, and 6.6 after 1, 5, 10, 20, and 30 CV cycles (Figure 11B and Table S5). Without Ni, the Fe–M_2_ bond length of monometallic FeO_*x*_ slightly decreases from 3.07 to 3.05 Å after 30 CV cycle treatment, while the CN slightly increases from 2.02 to 2.66 (Figure 11C and Table S5). The presence of Ni in the FeO_*x*_ shell facilitates the hydration of the shell, resulting in the drastic increase in the coordination of the Fe–M_2_ edge-sharing sites due to the evolution of the shell into the mixed Ni/Fe(OH)_*x*_ phase.^31,34,39,53,65^ On the other hand, the random interconnection of the edge-sharing hydroxide layers by corner-sharing FeO_6_ octahedra with an Fe–M_3_ bond at 3.46 Å decreases the plasticity (i.e., permeability) of the FeO_*x*_ shell.^64,66^ The increase in Fe–M_3_ coordination observed from 1.23 to 2.32 (after 30 CV cycles) and 2.55 (after 12 h of immersion in 1 M KOH) suggests an increase in corner-sharing FeO_6_ octahedra. The improvement of the structural rigidity prevents subsurface diffusion of OH^–^/H_2_O and exfoliation of edge-sharing FeO_6_-layered hydroxides,^66^ thereby better regulating the diffusion of OH^–^ into and Ni out of the nanostructures.

In addition to the above analysis, the potential effect of strains as a result of the core–shell interface should also be considered. The strain effect is typically confined to up to six atomic layers from the core–shell interface based on the recent studies of the Au-core Pd-shell catalysts.^67,68^ In our case, the thickness of the FeO_*x*_ shell is on average 2.4 nm. Given that 1 monolayer (ML) of FeO is approximately 0.3 nm,^69^ the FeO_*x*_ shell is about 8 ML thick. Thus, the strains at the core–shell interface will not affect the outmost surface of the shell where catalytic activity occurs. Further, we observed that CV cycling introduced a gap between the core and the shell as the shell became thicker and more porous, further alleviating any potential strains.

## Conclusions

We synthesized NiP_*x*_-FeO_*x*_ core–shell nanostructures for enhancing the OER and investigated their structural evolution during 30 CV cycling under 1 M KOH alkaline condition. Their OER activity increased with the increased number of cycling as indicated by the decrease in the overpotentials after 30 CV cycles at both 10 mV/cm^2^ (6 mV decrease) and 100 mV/cm^2^ (50 mV decrease). The increase in the OER activity was accompanied by the changes in the Ni^2+^/Ni^3+/4+^ redox region where the redox peak shifted to a lower potential and the redox current increased as the number of cycles increased, suggesting a structural evolution of the nanostructures. Based on the TEM and XPS results, we found that while the core–shell structure was retained, the core converted from NiP_*x*_ to Ni_3_(PO_4_)_2_ and Ni(OH)_2_ upon exposure to oxidative environments in alkaline media. During the conversion, more Ni integrated into the Fe shell to form the Ni/Fe(OH)_*x*_ surface that promoted the OER activity and led to the shifts of the Ni^2+^/Ni^3+/4+^ redox peak.

Further *ex situ* XAS investigation of the structural evolution was performed by monitoring the structural progression of the 30 CV cycles and comparing it with the core and shell materials alone. We found that the FeO_*x*_ shell could prevent excess diffusion of OH^–^ into the NiP_*x*_ core as a result of its increased structural rigidity and the presence of corner-sharing FeO_6_ octahedra. Since the NiP_*x*_ core is retained, the core–shell structures eventually reach an equilibrium between the two segregated Ni phases (i.e., the NiP_*x*_ core and the Ni/Fe(OH)_*x*_ shell) after about 20–25 CV cycles. Meanwhile, Ni/Fe(OH)_*x*_ at the surface could be replenished through the *in situ* migration of Ni from the core into the shell, which in the context of the OER, can potentially lead to improved stability. The work also lays the foundation for future studies of the reaction mechanisms on the complex structures by *in situ*/operando measurements coupled with theoretical modeling.^70^ The synthetic route for the FeO_*x*_ shell can be applied in other systems as a rational approach to controlling the structural rigidity and manipulation of the surface adsorption and ion diffusion of transition metal catalysts.

## Supporting Information Available

Ni^2+^/Ni^3+/4+^ oxidative region analysis; CV profiles of the core–shell nanoparticles; XRD and CV of NiP_*x*_ and FeO_*x*_; histograms of the size distribution of the core–shell nanoparticles; XPS elemental analysis on the ratios of Ni:Fe and Ni:P for samples; XPS of core–shell nanoparticles; balancing the NiP_*x*_-to-Ni(OH)_2_ conversion equation; LCF fits of the XAS spectra; Ni K-edge EXAFS fit results; Fe K-edge EXAFS fit results; real and imaginary components of the Ni K-edge EXAFS results; average ⟨Fe–O⟩ bond lengths; Ni and Fe K-edge *k*^3^-weighted FT EXAFS spectra; Ni and Fe path contributions of the Ni and Fe K-edge FT EXAFS fits (PDF)

## References

[ref1] Zeng K.; Zhang D. Recent Progress in Alkaline Water Electrolysis for Hydrogen Production and Applications. Prog. Energy Combust. Sci. 2010, 36, 307–326. 10.1016/j.pecs.2009.11.002.

[ref2] Ehlers J. C.; Feidenhans’l A. A.; Therkildsen K. T.; Larrazábal G. O. Affordable Green Hydrogen from Alkaline Water Electrolysis: Key Research Needs from an Industrial Perspective. ACS Energy Lett. 2023, 8, 1502–1509. 10.1021/acsenergylett.2c02897.

[ref3] Suen N.-T.; Hung S.-F.; Quan Q.; Zhang N.; Xu Y.-J.; Chen H. M. Electrocatalysis for the Oxygen Evolution Reaction: Recent Development and Future Perspectives. Chem. Soc. Rev. 2017, 46, 337–365. 10.1039/C6CS00328A. 28083578

[ref4] Seh Z. W.; Kibsgaard J.; Dickens C. F.; Chorkendorff I.; Nørskov J. K.; Jaramillo T. F. Combining Theory and Experiment in Electrocatalysis: Insights into Materials Design. Science 2017, 355, eaad4998 10.1126/science.aad4998. 28082532

[ref5] Dionigi F.; Strasser P. NiFe-Based (Oxy)Hydroxide Catalysts for Oxygen Evolution Reaction in Non-Acidic Electrolytes. Adv. Energy Mater. 2016, 6, 1600621 10.1002/aenm.201600621.

[ref6] Bodhankar P. M.; Sarawade P. B.; Singh G.; Vinu A.; Dhawale D. S. Recent Advances in Highly Active Nanostructured NiFe LDH Catalyst for Electrochemical Water Splitting. J. Mater. Chem. A 2021, 9, 3180–3208. 10.1039/D0TA10712C.

[ref7] Wygant B. R.; Kawashima K.; Mullins C. B. Catalyst or Precatalyst? The Effect of Oxidation on Transition Metal Carbide, Pnictide, and Chalcogenide Oxygen Evolution Catalysts. ACS Energy Lett. 2018, 3, 2956–2966. 10.1021/acsenergylett.8b01774.

[ref8] Dutta A.; Pradhan N. Developments of Metal Phosphides as Efficient OER Precatalysts. J. Phys. Chem. Lett. 2017, 8, 144–152. 10.1021/acs.jpclett.6b02249. 27981840

[ref9] Ray A.; Sultana S.; Paramanik L.; Parida K. Recent Advances in Phase, Size, and Morphology-Oriented Nanostructured Nickel Phosphide for Overall Water Splitting. J. Mater. Chem. A 2020, 8, 19196–19245. 10.1039/D0TA05797E.

[ref10] Dutta A.; Mutyala S.; Samantara A. K.; Bera S.; Jena B. K.; Pradhan N. Synergistic Effect of Inactive Iron Oxide Core on Active Nickel Phosphide Shell for Significant Enhancement in Oxygen Evolution Reaction Activity. ACS Energy Letters 2018, 3, 141–148. 10.1021/acsenergylett.7b01141.

[ref11] Kang Q.; Lai D.; Tang W.; Lu Q.; Gao F. Intrinsic Activity Modulation and Structural Design of NiFe Alloy Catalysts for an Efficient Oxygen Evolution Reaction. Chem. Sci. 2021, 12, 3818–3835. 10.1039/D0SC06716D. 34163652 PMC8179442

[ref12] Zhou Y.; Fan H. J. Progress and Challenge of Amorphous Catalysts for Electrochemical Water Splitting. ACS Mater. Lett. 2021, 3, 136–147. 10.1021/acsmaterialslett.0c00502.

[ref13] Chen G.; Zhu Y.; Chen H. M.; Hu Z.; Hung S.; Ma N.; Dai J.; Lin H.; Chen C.; Zhou W.; Shao Z. An Amorphous Nickel–Iron-Based Electrocatalyst with Unusual Local Structures for Ultrafast Oxygen Evolution Reaction. Adv. Mater. 2019, 31, 1900883 10.1002/adma.201900883. 31099042

[ref14] Gao L.; Cui X.; Sewell C. D.; Li J.; Lin Z. Recent Advances in Activating Surface Reconstruction for the High-Efficiency Oxygen Evolution Reaction. Chem. Soc. Rev. 2021, 50, 8428–8469. 10.1039/D0CS00962H. 34259239

[ref15] Saruyama M.; Kim S.; Nishino T.; Sakamoto M.; Haruta M.; Kurata H.; Akiyama S.; Yamada T.; Domen K.; Teranishi T. Phase-Segregated NiP_*x*_@ FeP_*y*_O_*z*_ Core@ Shell Nanoparticles: Ready-to-Use Nanocatalysts for Electro-and Photo-Catalytic Water Oxidation through *in situ* Activation by Structural Transformation and Spontaneous Ligand Removal. Chem. Sci. 2018, 9, 4830–4836. 10.1039/C8SC00420J. 29910935 PMC5982198

[ref16] Ma X.; Chang Y.; Zhang Z.; Tang J. Forest-Like NiCoP@Cu_3_P Supported on Copper Foam as a Bifunctional Catalyst for Efficient Water Splitting. J. Mater. Chem. A 2018, 6, 2100–2106. 10.1039/C7TA09619D.

[ref17] Manso R. H.; Acharya P.; Deng S.; Crane C. C.; Reinhart B.; Lee S.; Tong X.; Nykypanchuk D.; Zhu J.; Zhu Y.; Greenlee L. F.; Chen J. Controlling the 3-D Morphology of Ni–Fe-based Nanocatalysts for the Oxygen Evolution Reaction. Nanoscale 2019, 11, 8170–8184. 10.1039/C8NR10138H. 30775739

[ref18] Acharya P.; Manso R. H.; Hoffman A. S.; Bakovic S. I. P.; Kékedy-Nagy L.; Bare S. R.; Chen J.; Greenlee L. F. Fe Coordination Environment, Fe-Incorporated Ni(OH)_2_ Phase, and Metallic Core Are Key Structural Components to Active and Stable Nanoparticle Catalysts for the Oxygen Evolution Reaction. ACS Catal. 2022, 12, 1992–2008. 10.1021/acscatal.1c04881.

[ref19] Liu X.; Meng J.; Zhu J.; Huang M.; Wen B.; Guo R.; Mai L. Comprehensive Understandings into Complete Reconstruction of Precatalysts: Synthesis, Applications, and Characterizations. Adv. Mater. 2021, 33, 2007344 10.1002/adma.202007344. 34050565

[ref20] Trotochaud L.; Young S. L.; Ranney J. K.; Boettcher S. W. Nickel–Iron Oxyhydroxide Oxygen-Evolution Electrocatalysts: The Role of Intentional and Incidental Iron Incorporation. J. Am. Chem. Soc. 2014, 136, 6744–6753. 10.1021/ja502379c. 24779732

[ref21] Ravel B.; Newville M. Athena, Artemis, Hephaestus: Data Analysis for X-Ray Absorption Spectroscopy Using IFEFFIT. J. Synchrotron. Radiat. 2005, 12, 537–41. 10.1107/S0909049505012719. 15968136

[ref22] Ramesh T.; Kamath P. V.; Shivakumara C. Classification of Stacking Faults and Their Stepwise Elimination During the Disorder⃗ Order Transformation of Nickel Hydroxide. Acta Crystallogr. B. Struct. Sci. 2006, 62, 530–536. 10.1107/S0108768106013188. 16840802

[ref23] Kazimirov V. Y.; Smirnov M. B.; Bourgeois L.; Guerlou-Demourgues L.; Servant L.; Balagurov A. M.; Natkaniec I.; Khasanova N. R.; Antipov E. V. Atomic Structure and Lattice Dynamics of Ni and Mg Hydroxides. Solid State Ionics 2010, 181, 1764–1770. 10.1016/j.ssi.2010.10.002.

[ref24] Fruchart R.; Roger A.; Senateur J. P. Crystallographic and Magnetic Properties of Solid Solutions of the Phosphides M_2_P, M = Cr, Mn, Fe, Co, and Ni. J. Appl. Phys. 1969, 40, 1250–1257. 10.1063/1.1657617.

[ref25] Calvo C.; Faggiani R. Structure of Nickel Orthophosphate. Can. J. Chem. 1975, 53, 1516–1520. 10.1139/v75-210.

[ref26] Wyckoff R. Lepidocrocite Structure. Cryst. Struct. 1963, 1, 290–295.

[ref27] Blake R.; Hessevick R.; Zoltai T.; Finger L. W. Refinement of the Hematite Structure. Am. Mineral. 1966, 51, 123–129.

[ref28] Okamoto Y.; Nitta Y.; Imanaka T.; Teranishi S. Surface Characterisation of Nickel Boride and Nickel Phosphide Catalysts by X-Ray Photoelectron Spectroscopy. J. Chem. Soc., Faraday Trans. 1 1979, 75, 2027–2039. 10.1039/f19797502027.

[ref29] Yamamoto T. Assignment of Pre-Edge Peaks in K-Edge X-Ray Absorption Spectra of 3d Transition Metal Compounds: Electric Dipole or Quadrupole?. Xray Spectrom. 2008, 37, 572–584. 10.1002/xrs.1103.

[ref30] Mansour A.; Melendres C.; Pankuch M.; Brizzolara R. X-Ray Absorption Fine Structure Spectra and the Oxidation State of Nickel in Some of Its Oxycompounds. J. Electrochem. Soc. 1994, 141, L69 10.1149/1.2054990.

[ref31] Balasubramanian M.; Melendres C. A.; Mini S. X-Ray Absorption Spectroscopy Studies of the Local Atomic and Electronic Structure of Iron Incorporated into Electrodeposited Hydrous Nickel Oxide Films. J. Phys. Chem. B 2000, 104, 4300–4306. 10.1021/jp9921710.

[ref32] Westre T. E.; Kennepohl P.; DeWitt J. G.; Hedman B.; Hodgson K. O.; Solomon E. I. A Multiplet Analysis of Fe K-Edge 1s → 3d Pre-Edge Features of Iron Complexes. J. Am. Chem. Soc. 1997, 119, 6297–6314. 10.1021/ja964352a.

[ref33] Boubnov A.; Lichtenberg H.; Mangold S.; Grunwaldt J. D. Identification of the Iron Oxidation State and Coordination Geometry in Iron Oxide- and Zeolite-Based Catalysts Using Pre-Edge XAS Analysis. J. Synchrotron. Radiat. 2015, 22, 410–26. 10.1107/S1600577514025880. 25723943

[ref34] Abbott D. F.; Fabbri E.; Borlaf M.; Bozza F.; Schäublin R.; Nachtegaal M.; Graule T.; Schmidt T. J. Operando X-Ray Absorption Investigations into the Role of Fe in the Electrochemical Stability and Oxygen Evolution Activity of Ni_1-x_Fe_x_O_y_ Nanoparticles. J. Mater. Chem. A 2018, 6, 24534–24549. 10.1039/C8TA09336A.

[ref35] Baker M. L.; Mara M. W.; Yan J. J.; Hodgson K. O.; Hedman B.; Solomon E. I. K- and L-Edge X-Ray Absorption Spectroscopy (XAS) and Resonant Inelastic X-Ray Scattering (RIXS) Determination of Differential Orbital Covalency (DOC) of Transition Metal Sites. Coord. Chem. Rev. 2017, 345, 182–208. 10.1016/j.ccr.2017.02.004. 28970624 PMC5621773

[ref36] Kim S.; Tryk D. A.; Antonio M. R.; Carr R.; Scherson D. In Situ X-Ray Absorption Fine Structure Studies of Foreign Metal Ions in Nickel Hydrous Oxide Electrodes in Alkaline Electrolytes. J. Phys. Chem. 1994, 98, 10269–10276. 10.1021/j100091a049.

[ref37] McBreen J.; O’Grady W. E.; Tourillon G.; Dartyge E.; Fontaine A.; Pandya K. I. In Situ Time-Resolved X-Ray Absorption near Edge Structure Study of the Nickel Oxide Electrode. J. Phys. Chem. 1989, 93, 6308–6311. 10.1021/j100354a010.

[ref38] Klaus S.; Louie M. W.; Trotochaud L.; Bell A. T. Role of Catalyst Preparation on the Electrocatalytic Activity of Ni_1–x_Fe_x_OOH for the Oxygen Evolution Reaction. J. Phys. Chem. C 2015, 119, 18303–18316. 10.1021/acs.jpcc.5b04776.

[ref39] Friebel D.; Louie M. W.; Bajdich M.; Sanwald K. E.; Cai Y.; Wise A. M.; Cheng M.-J.; Sokaras D.; Weng T.-C.; Alonso-Mori R.; Davis R. C.; Bargar J. R.; Nørskov J. K.; Nilsson A.; Bell A. T. Identification of Highly Active Fe Sites in (Ni, Fe) OOH for Electrocatalytic Water Splitting. J. Am. Chem. Soc. 2015, 137, 1305–1313. 10.1021/ja511559d. 25562406

[ref40] Klaus S.; Cai Y.; Louie M. W.; Trotochaud L.; Bell A. T. Effects of Fe Electrolyte Impurities on Ni(OH)_2_/NiOOH Structure and Oxygen Evolution Activity. J. Phys. Chem. C 2015, 119, 7243–7254. 10.1021/acs.jpcc.5b00105.

[ref41] Hu M.; Lei L. Effects of Particle Size on the Electrochemical Performances of a Layered Double Hydroxide,[Ni_4_Al(OH)_10_] NO_3_. J. Solid State Electrochem. 2007, 11, 847–852. 10.1007/s10008-006-0231-y.

[ref42] Shukla A.; Ravikumar M.; Balasubramanian T. Nickel/Iron Batteries. J. Power Sources 1994, 51, 29–36. 10.1016/0378-7753(94)01953-3.

[ref43] Hall D. S.; Lockwood D. J.; Bock C.; MacDougall B. R. Nickel Hydroxides and Related Materials: A Review of Their Structures, Synthesis and Properties. Proc. Math. Phys. Eng. Sci. 2015, 471, 20140792 10.1098/rspa.2014.0792. 25663812 PMC4309132

[ref44] Młynarek G.; Paszkiewicz M.; Radniecka A. The Effect of Ferric Ions on the Behaviour of a Nickelous Hydroxide Electrode. J. Appl. Electrochem. 1984, 14, 145–149. 10.1007/BF00618733.

[ref45] Vandichel M.; Laasonen K.; Kondov I. Oxygen Evolution and Reduction on Fe-Doped Niooh: Influence of Solvent, Dopant Position and Reaction Mechanism. Top. Catal. 2020, 63, 833–845. 10.1007/s11244-020-01334-8.

[ref46] O’day P. A.; Rivera N. Jr; Root R.; Carroll S. A. X-Ray Absorption Spectroscopic Study of Fe Reference Compounds for the Analysis of Natural Sediments. Am. Mineral. 2004, 89, 572–585. 10.2138/am-2004-0412.

[ref47] Fujita S.; Yamaguchi S.; Yamazoe S.; Yamasaki J.; Mizugaki T.; Mitsudome T. Nickel Phosphide Nanoalloy Catalyst for the Selective Deoxygenation of Sulfoxides to Sulfides under Ambient H_2_ Pressure. Org. Biomol. Chem. 2020, 18, 8827–8833. 10.1039/D0OB01603A. 33179696

[ref48] Gubbins K. E.; Walker R. D. The Solubility and Diffusivity of Oxygen in Electrolytic Solutions. J. Electrochem. Soc. 1965, 112, 469 10.1149/1.2423575.

[ref49] Moreau L. M.; Ha D.-H.; Bealing C. R.; Zhang H.; Hennig R. G.; Robinson R. D. Unintended Phosphorus Doping of Nickel Nanoparticles During Synthesis with Top: A Discovery through Structural Analysis. Nano Lett. 2012, 12, 4530–4539. 10.1021/nl301642g. 22845819

[ref50] Moreau L. M.; Ha D.-H.; Zhang H.; Hovden R.; Muller D. A.; Robinson R. D. Defining Crystalline/Amorphous Phases of Nanoparticles through X-Ray Absorption Spectroscopy and X-Ray Diffraction: The Case of Nickel Phosphide. Chem. Mater. 2013, 25, 2394–2403. 10.1021/cm303490y.

[ref51] Hu F.; Zhu S.; Chen S.; Li Y.; Ma L.; Wu T.; Zhang Y.; Wang C.; Liu C.; Yang X.; Song L.; Yang X.; Xiong Y. Amorphous Metallic NiFeP: A Conductive Bulk Material Achieving High Activity for Oxygen Evolution Reaction in Both Alkaline and Acidic Media. Adv. Mater. 2017, 29, 1606570 10.1002/adma.201606570. 28639333

[ref52] Görlin M.; Chernev P.; Ferreira de Araújo J.; Reier T.; Dresp S. r.; Paul B.; Krähnert R.; Dau H.; Strasser P. Oxygen Evolution Reaction Dynamics, Faradaic Charge Efficiency, and the Active Metal Redox States of Ni–Fe Oxide Water Splitting Electrocatalysts. J. Am. Chem. Soc. 2016, 138, 5603–5614. 10.1021/jacs.6b00332. 27031737

[ref53] González-Flores D.; Klingan K.; Chernev P.; Loos S.; Mohammadi M. R.; Pasquini C.; Kubella P.; Zaharieva I.; Smith R. D. L.; Dau H. Nickel-Iron Catalysts for Electrochemical Water Oxidation–Redox Synergism Investigated by *in Sstu* X-Ray Spectroscopy with Millisecond Time Resolution. Sustain. Energy Fuels 2018, 2, 1986–1994. 10.1039/C8SE00114F.

[ref54] Liu P. F.; Li X.; Yang S.; Zu M. Y.; Liu P.; Zhang B.; Zheng L. R.; Zhao H.; Yang H. G. Ni_2_P(O)/Fe_2_P(O) Interface Can Boost Oxygen Evolution Electrocatalysis. ACS Energy Lett. 2017, 2, 2257–2263. 10.1021/acsenergylett.7b00638.

[ref55] Pandya K. I.; O’Grady W. E.; Corrigan D. A.; McBreen J.; Hoffman R. W. Extended X-Ray Absorption Fine Structure Investigations of Nickel Hydroxides. J. Phys. Chem. 1990, 94, 21–26. 10.1021/j100364a005.

[ref56] Lee S.-Y.; Kim I.-S.; Cho H.-S.; Kim C.-H.; Lee Y.-K. Resolving Potential-Dependent Degradation of Electrodeposited Ni(OH)_2_ Catalysts in Alkaline Oxygen Evolution Reaction (OER): *In situ* XANES Studies. Appl. Catal. B: Environ. 2021, 284, 119729 10.1016/j.apcatb.2020.119729.

[ref57] Wilke M.; Farges F.; Petit P.-E.; Brown G. E. Jr; Martin F. Oxidation State and Coordination of Fe in Minerals: An Fe K-XANES Spectroscopic Study. Am. Mineral. 2001, 86, 714–730. 10.2138/am-2001-5-612.

[ref58] Fdez-Gubieda M. L.; García-Prieto A.; Alonso J.; Meneghini C. X-Ray Absorption Fine Structure Spectroscopy in Fe Oxides and Oxyhydroxides, Faivre D., Ed., In Iron Oxides, 2016, 397–422

[ref59] Combes J.; Manceau A.; Calas G. Formation of Ferric Oxides from Aqueous Solutions: A Polyhedral Approach by X-Ray Absorption Spectroscopy: II. Hematite Formation from Ferric Gels. Geochim. et Cosmochim. Acta 1990, 54, 1083–1091. 10.1016/0016-7037(90)90440-V.

[ref60] Manceau A.; Drits V. Local Structure of Ferrihydrite and Feroxyhite by EXAFS Spectroscopy. Clay Miner. 1993, 28, 165–184. 10.1180/claymin.1993.028.2.01.

[ref61] Drits V.; Sakharov B.; Salyn A.; Manceau A. Structural Model for Ferrihydrite. Clay Miner. 1993, 28, 185–207. 10.1180/claymin.1993.028.2.02.

[ref62] Maillot F.; Morin G.; Wang Y.; Bonnin D.; Ildefonse P.; Chaneac C.; Calas G. New Insight into the Structure of Nanocrystalline Ferrihydrite: EXAFS Evidence for Tetrahedrally Coordinated Iron (III). Geochim. Cosmochim. Acta 2011, 75, 2708–2720. 10.1016/j.gca.2011.03.011.

[ref63] Corrias A.; Ennas G.; Mountjoy G.; Paschina G. An X-Ray Absorption Spectroscopy Study of the Fe K Edge in Nanosized Maghemite and in Fe_2_O_3_–SiO_2_ Nanocomposites. Phys. Chem. Chem. Phys. 2000, 2, 1045–1050. 10.1039/a908698f.

[ref64] Manceau A. Critical Evaluation of the Revised Akdalaite Model for Ferrihydrite. Am. Mineral. 2011, 96, 521–533. 10.2138/am.2011.3583.

[ref65] Anantharaj S.; Kundu S.; Noda S. “The Fe Effect”: A Review Unveiling the Critical Roles of Fe in Enhancing Oer Activity of Ni and Co Based Catalysts. Nano Energy 2021, 80, 105514 10.1016/j.nanoen.2020.105514.

[ref66] Hu B.; Yan X.; Wang W.; Li Y.; Li H.; Hong M.; Liu F.; Yin H. Iron Oxyhydroxide Polytype (γ-, δ-and β-FeOOH) Structures Govern Zn Mobility. Chem. Geol. 2022, 614, 121167 10.1016/j.chemgeo.2022.121167.

[ref67] van der Hoeven J. E. S.; Jelic J.; Olthof L. A.; Totarella G.; van Dijk-Moes R. J. A.; Krafft J.-M.; Louis C.; Studt F.; van Blaaderen A.; de Jongh P. E. Unlocking Synergy in Bimetallic Catalysts by Core–Shell Design. Nat. Mater. 2021, 20, 1216–1220. 10.1038/s41563-021-00996-3. 33958769

[ref68] Zhang X.; et al. Conjugated Dual Size Effect of Core-Shell Particles Synergizes Bimetallic Catalysis. Nat. Commun. 2023, 14, 530 10.1038/s41467-023-36147-2. 36725854 PMC9892500

[ref69] Weiss W.; Somorjai G. A. Preparation and Structure of 1–8 Monolayer Thick Epitaxial Iron Oxide Films Grown on Pt(111). J. Vac. Sci. Technol. A 1993, 11, 2138–2144. 10.1116/1.578382.

[ref70] Zhu K.; Zhu X.; Yang W. Application of *in situ* Techniques for the Characterization of NiFe-Based Oxygen Evolution Reaction (OER) Electrocatalysts. Angew. Chem., Int. Ed. 2019, 58, 1252–1265. 10.1002/anie.201802923. 29665168

