# Supplementary material for: Revealing Structural Evolution of Nickel Phosphide-Iron Oxide Core–Shell Nanocatalysts in Alkaline Medium for the Oxygen Evolution Reaction
Source: Chem Mater. 2024 Jun 21;36(13):6440–53. doi: 10.1021/acs.chemmater.4c00379 (PMC11238331; doi:10.1021/acs.chemmater.4c00379)
Supplement: Supplementary file 1 — cm4c00379_si_001.pdf [file cm4c00379_si_001.pdf]

Supporting Information

Revealing Structural Evolution of Nickel Phosphide-Iron Oxide Core-Shell Nanocatalysts in Alkaline Medium for the Oxygen Evolution Reaction

Ryan H. Manso[a], Jiyun Hong[b], Wei Wang[c], Prashant Acharya[d], Adam S. Hoffman[b], Xiao Tong[e], Feng Wang[a], Lauren F. Greenlee[d,f], Yimei Zhu[c], Simon R. Bare[b], and Jingyi Chen[a*]

[a]*Department of Chemistry and Biochemistry, University of Arkansas, Fayetteville, AR 72701, United States*

[b]*Stanford Synchrotron Radiation Lightsource, SLAC National Accelerator Laboratory, Menlo Park, CA 94025, United States*

[c]*Condensed Matter Physics and Materials Science Department, Brookhaven National Laboratory, Upton, NY 11973, United States*

[d]*Ralph E. Martin Department of Chemical Engineering, University of Arkansas, Fayetteville, AR 72701, United States*

[e]*Center for Functional Nanomaterials, Brookhaven National Laboratory, Upton, NY 11973, United States*

[f]*Department of Chemical Engineering, Pennsylvania State University, University Park, PA, 16802, United States*

[*]Email: chenj@uark.edu

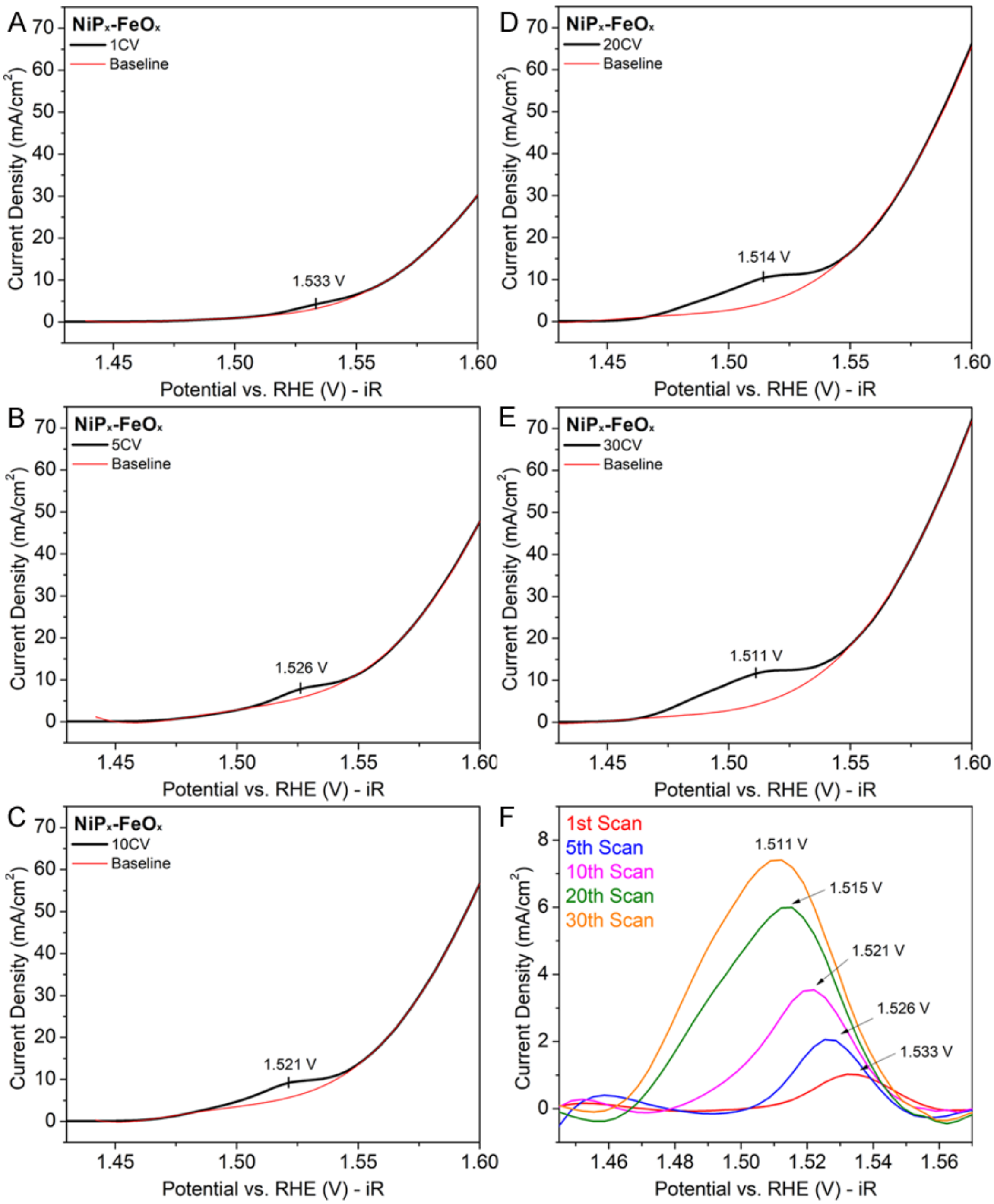

Figure S1. (A-E) $Ni^{2+}/Ni^{3+/4+}$ oxidative region and their baseline fits after cycling: (A) 1; (B) 5; (C) 10; (D) 20; and (E) 30 CV cycles. (F) The baseline-subtracted $Ni^{2+}/Ni^{3+/4+}$ oxidation curves that accounted for the OER contribution with their peak positions labeled.

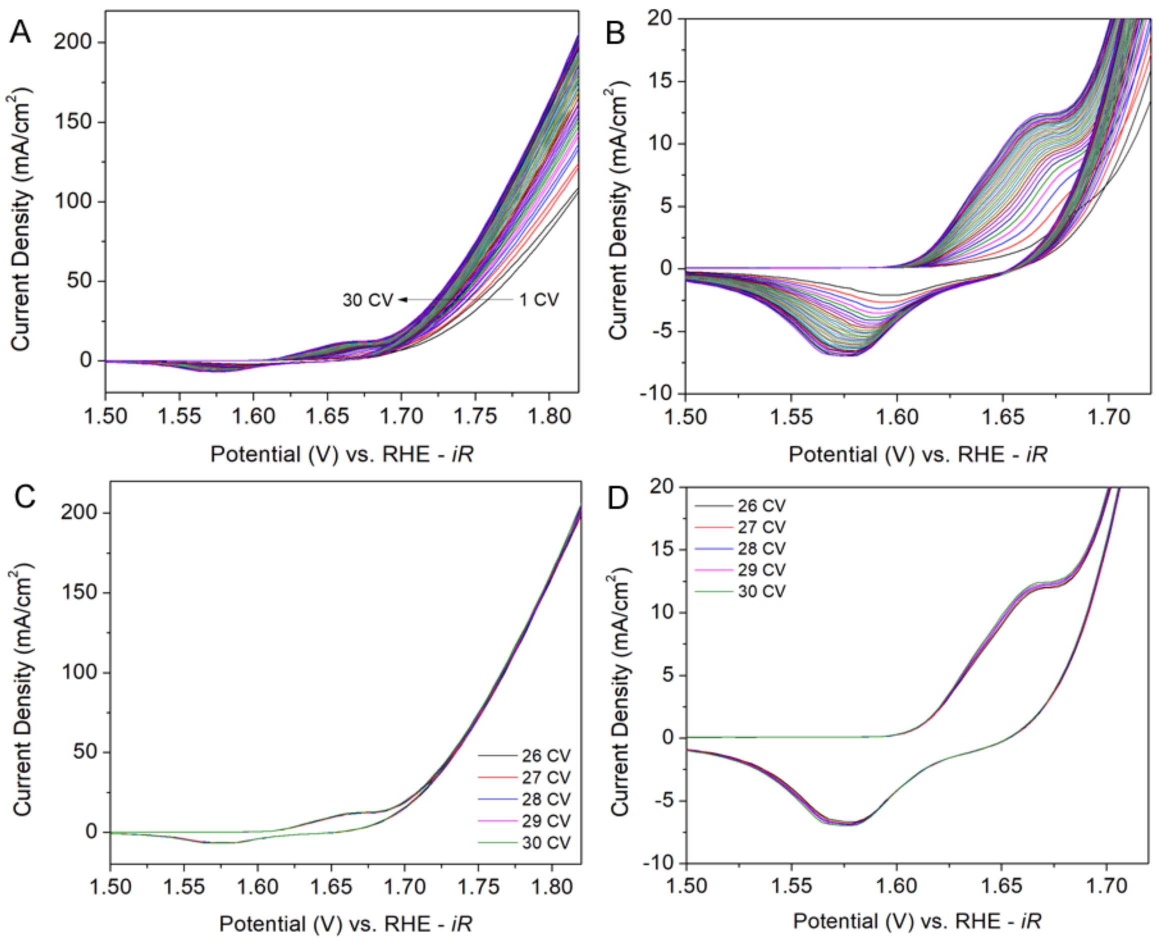

Figure S2. CV profiles of the NiP_x-FeO_x nanoparticles of 30 cycles at a scan rate of 10 mV/s from 1.0 – 1.8 V vs RHE in 1 M KOH: (A,B) all 30 scans and (C,D) 26 – 30 scans.

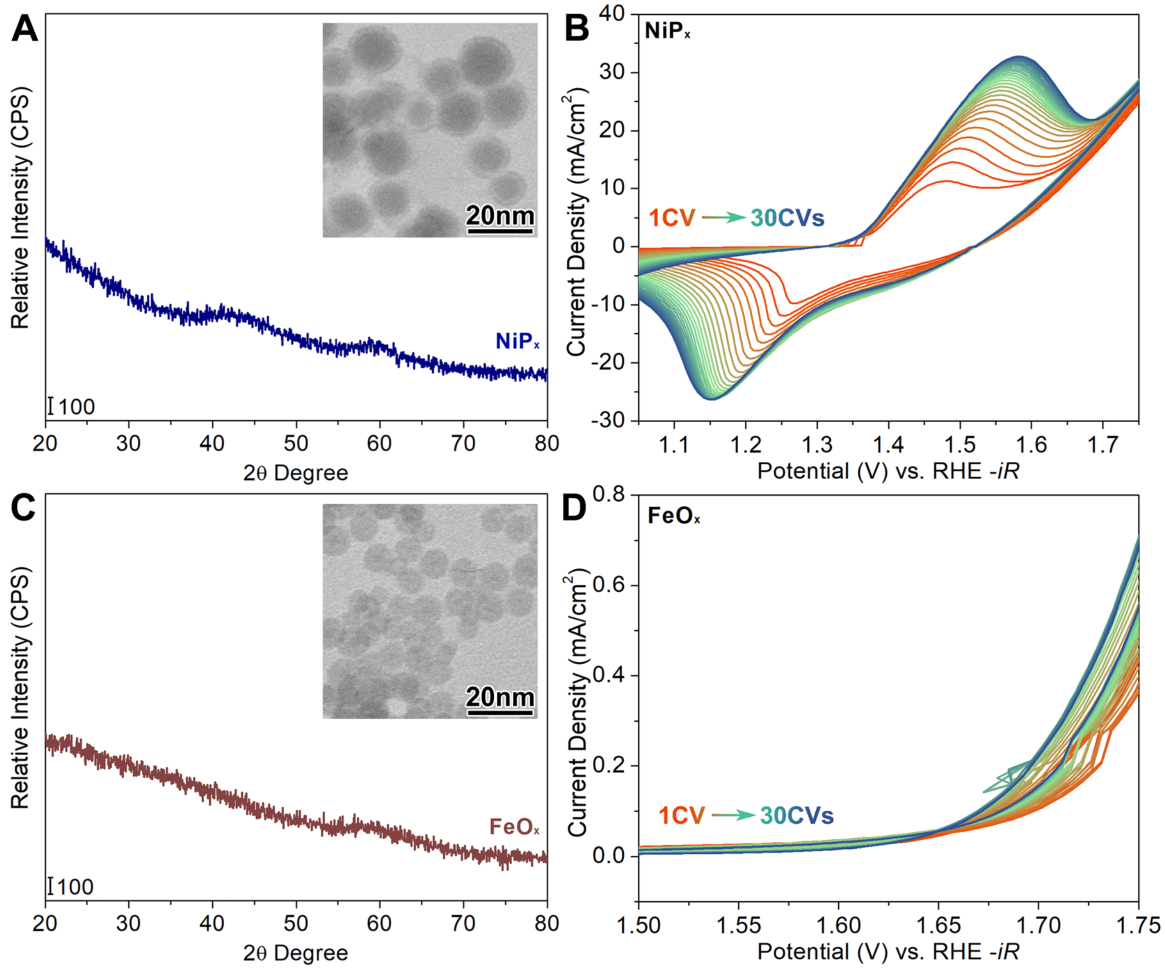

Figure S3. (A) XRD of NiP_x nanoparticles with a TEM image as the insert; (B) CV profiles of the NiP_x nanoparticles of 30 cycles at a scan rate of 10 mV/s from 1.0 – 1.8 V vs RHE in 1 M KOH; (C) XRD of FeO_x nanoparticles with a TEM image as the insert; and (D) CV profiles of the FeO_x nanoparticles of 30 cycles at a scan rate of 10 mV/s from 1.0 – 1.8 V vs RHE in 1 M KOH.

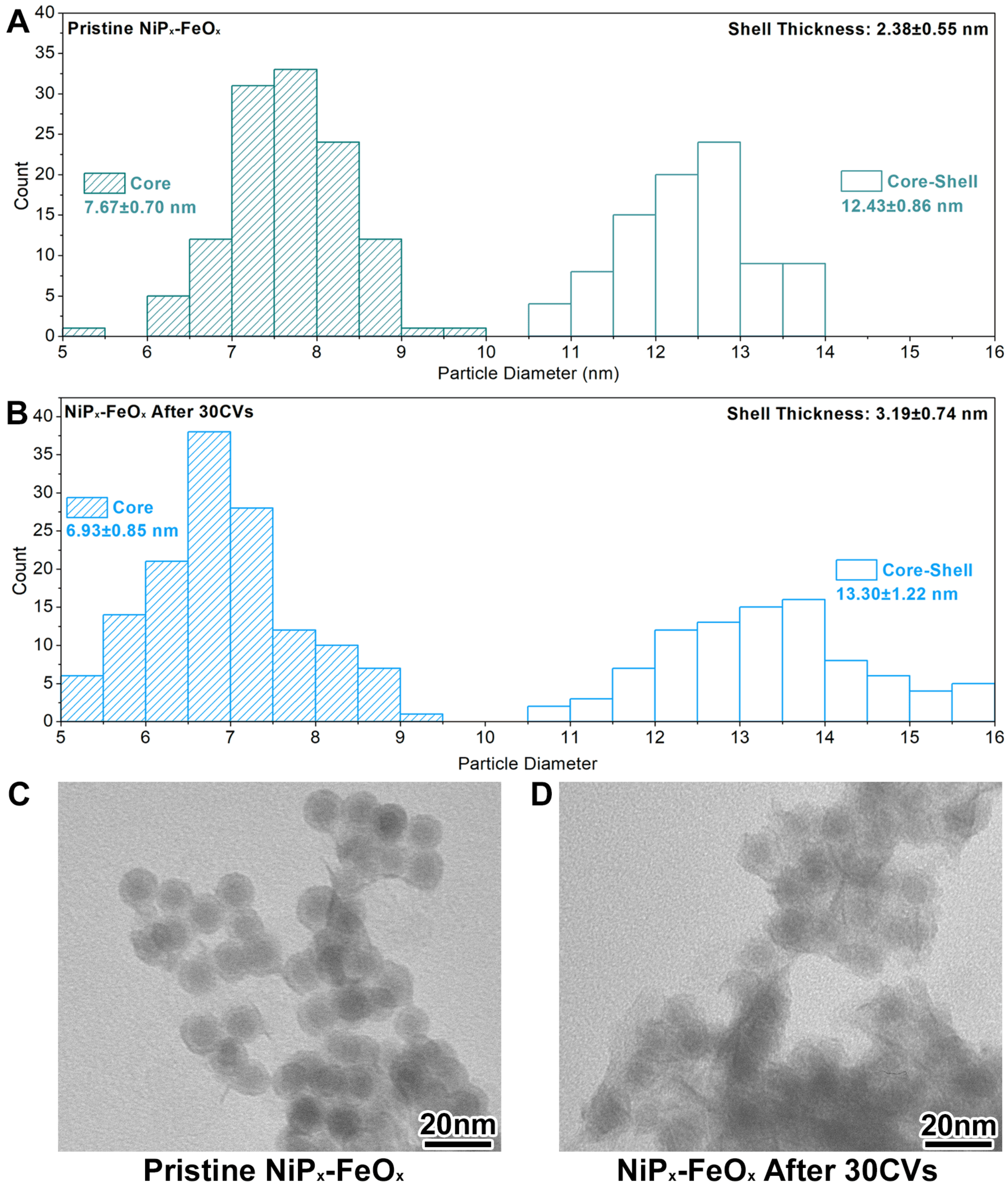

Figure S4. Histograms of the size distribution of the NiP_x-FeO_x core-shell nanoparticles before (A) and after (B) 30 CV cycles with TEM images of the corresponding (C) pristine and (D) 30 CV-treated nanoparticles.

Table S1. XPS elemental analysis on the ratios of Ni:Fe and Ni:P for samples: (A) Pristine (as-synthesized) NiP_x-FeO_x; (B) NiP_x-FeO_x after 30 CVs, and (C) NiP_x-FeO_x after 12 h KOH soaking.

(A) Pristine NiP_x-FeO_x

Analysis	Ni 2p	P 2p	Fe 2p	Ni:P	Ni:Fe
Area	22368.8	625.7	14504.7	-	-
R.S.F.	22.18	1.192	16.42	-	-
Relative Intensity	1008.5	524.9	883.4	-	-
Element Ratio	-	-	-	1.92	1.14

(B) NiP_x-FeO_x after 30 CVs

Analysis	Ni 2p	P 2p	Fe 2p	Ni:P	Ni:Fe
Area	21122.4	304.4	12752.9	-	-
R.S.F.	22.18	1.192	16.42	-	-
Relative Intensity	952.3	255.4	776.7	-	-
Element Ratio	-	-	-	3.73	1.23

(C) NiP_x-FeO_x after 12 h KOH soaking

Analysis	Ni 2p	P 2p	Fe 2p	Ni:P	Ni:Fe
area	26780.4	162.1	13155.5	-	-
R.S.F.	22.18	1.192	16.42	-	-
Relative Intensity	1207.4	136.0	801.2	-	-
Element Ratio	-	-	-	8.88	1.51

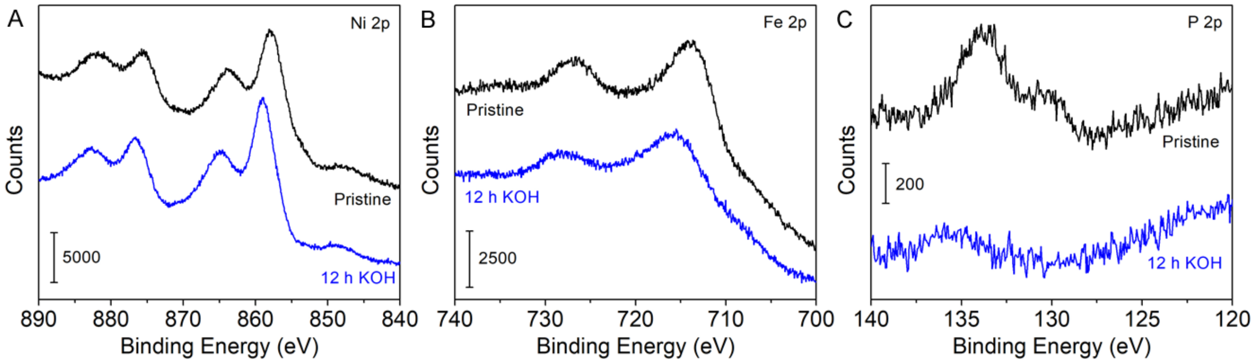

Figure S5. XPS spectra of Ni 2p (A), Fe 2p (B), and P 2p (C) of the core-shell nanoparticles after 1 M KOH treatment for 12 h.

Balance the chemical reaction of dNiP$_{0.5}$ + eH$_2$O + fO$_2$ → aNi$_3$(PO$_4$)$_2$ + bPH$_3$ + cNi(OH)$_2$.
The stoichiometric relationship of the chemical reaction was solved based on mass conservation of Ni, P, H, and O using linear algebra. A set of stoichiometric coefficient *a*, *b*, *c*, *d*, *e*, and *f* for each substance was expressed in eq. 1:

$$dNiP_{0.5} + eH_2O + fO_2 \rightarrow aNi_3(PO_4)_2 + bPH_3 + cNi(OH)_2 \quad (1)$$

To balance the eq. 1, the mass of each substance must be conserved. We then establish the following four equations, eq. 2-5:

$$\text{Ni:} \quad d = 3a + c \quad (2)$$

$$\text{P:} \quad 0.5d = 2a + b \quad (3)$$

$$\text{H:} \quad 2e = 3b + 2c \quad (4)$$

$$\text{O:} \quad e + 2f = 8a + 2c \quad (5)$$

With 4 equations, multiple solutions exist for the stoichiometry coefficients that are redundant. We set *d* to one. And solve the coefficients *a, b, c*, and *e* as a function of the oxygen coefficient *f*. This allows us to model the variation of product distribution as O_2 partial pressure changes.

$$a = \frac{4f-0.5}{16} \qquad c = 1.00 - \frac{12f-1.5}{16}$$

$$b = 0.50 - \frac{8f-1}{16} \qquad e = 1.75 - \frac{12f-1.5}{8}$$

a, b, c and *e* gives the stoichiometries of $Ni_3(PO_4)_2$, PH_3, and $Ni(OH)_2$ at different *f* values. The result was plotted in **Figure 4B**.

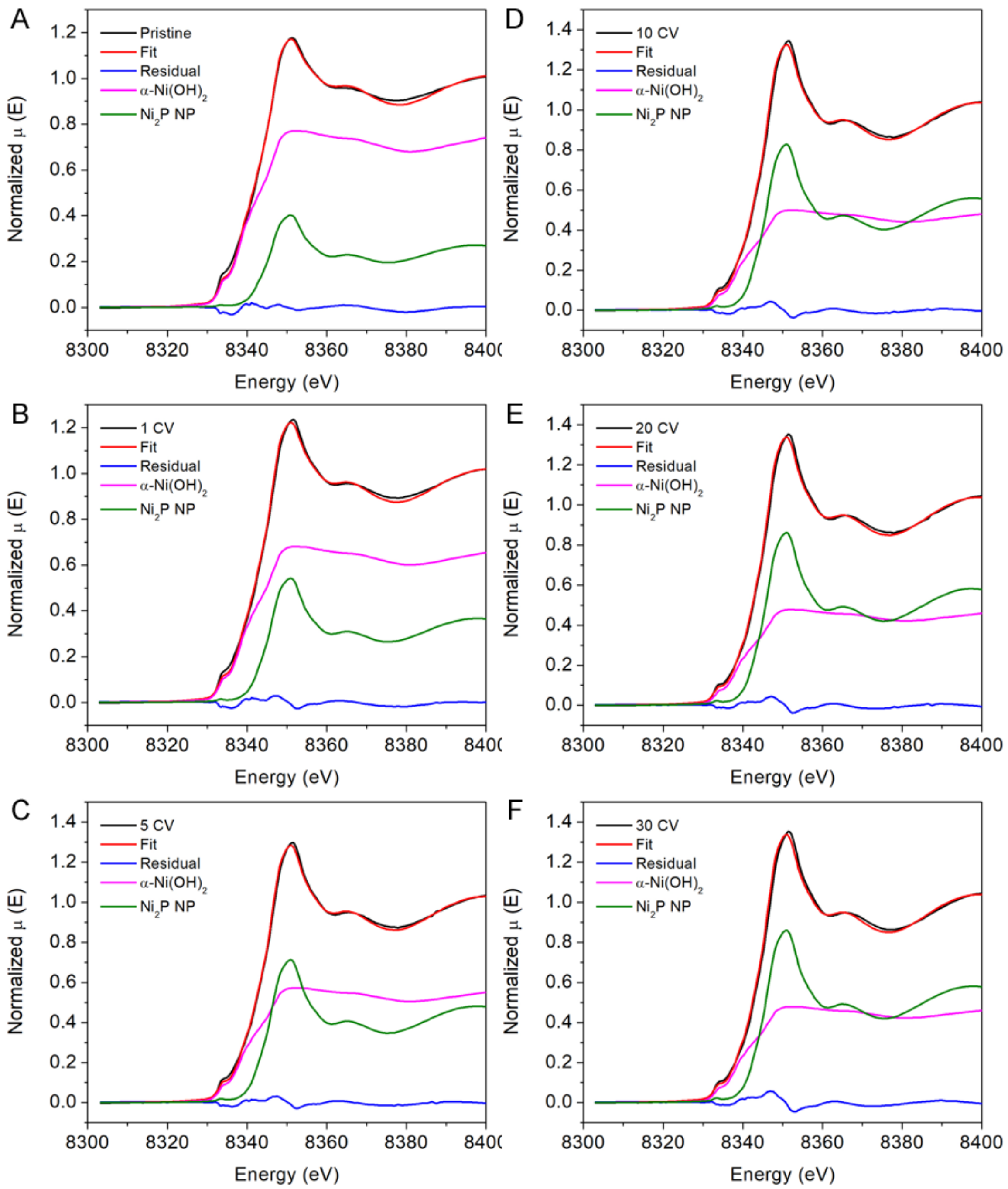

Figure S6. LCF analysis of pristine sample (A) and after 1 (B), 5 (C), 10 (D), 20 (E), and 30 (F) CV cycles using Ni_2P and α-$Ni(OH)_2$ XAS data.

Table S4. Ni K-edge EXAFS fit results for the pristine, 1, 5, 10, 20, 30 CV, and KOH-treated NiP_x-FeO_x nanocatalyst; and for the pristine and 30CV-treated NiP_x monometallic nanostructure.

Sample	Path	Shell	R(Å)	CN	σ^2	E_o (eV)	χ^2	R-factor
Pristine NiP_x-FeO_x	α-$Ni(OH)_2$	*Ni-O*	2.07±0.06	4.0±2.1	0.013±0.008	-5.9±1.4	123.2	0.00298
	Ni_2P	*Ni-P_1*	2.26±0.01	2.6±1.1	0.010±0.003			
	Ni_2P	*Ni-Ni*	2.55±0.02	2.1±0.6	0.010±0.003			
	α-$Ni(OH)_2$	*Ni-M_1*	3.08±0.01	1.8±0.5	0.010±0.003			
1CV NiP_x-FeO_x	α-$Ni(OH)_2$	*Ni-O*	2.06±0.04	4.1±1.4	0.011±0.005	-5.7±1.0	64.6	0.00380
	Ni_2P	*Ni-P_1*	2.26±0.02	1.9±0.6	0.008±0.002			
	Ni_2P	*Ni-Ni*	2.55±0.01	1.7±0.3	0.008±0.002			
	α-$Ni(OH)_2$	*Ni-M_1*	3.08±0.01	2.4±0.5	0.008±0.002			
5CV NiP_x-FeO_x	α-$Ni(OH)_2$	*Ni-O*	2.07±0.03	5.3±1.4	0.012±0.003	-4.6±1.0	45.2	0.00652
	Ni_2P	*Ni-P_1*	2.25±0.01	1.6±0.6	0.007±0.002			
	Ni_2P	*Ni-Ni*	2.56±0.01	1.5±0.2	0.007±0.002			
	α-$Ni(OH)_2$	*Ni-M_1*	3.08±0.01	2.8±0.5	0.007±0.002			
10CV NiP_x-FeO_x	α-$Ni(OH)_2$	*Ni-O*	2.06±0.03	5.7±1.6	0.011±0.004	-4.2±0.8	50.6	0.00289
	Ni_2P	*Ni-P_1*	2.25±0.03	1.4±0.9	0.008±0.002			
	Ni_2P	*Ni-Ni*	2.56±0.01	1.4±0.2	0.008±0.002			
	α-$Ni(OH)_2$	*Ni-M_1*	3.08±0.01	3.5±0.6	0.008±0.002			
20CV NiP_x-FeO_x	α-$Ni(OH)_2$	*Ni-O*	2.07±0.03	6.1±1.5	0.010±0.002	-3.6±0.7	41.2	0.00357
	Ni_2P	*Ni-P_1*	2.24±0.02	1.4±0.9	0.008±0.001			
	Ni_2P	*Ni-Ni*	2.57±0.01	1.4±0.2	0.008±0.001			
	α-$Ni(OH)_2$	*Ni-M_1*	3.09±0.01	3.4±0.4	0.008±0.001			
30CV NiP_x-FeO_x	$Ni(OH)_2$	*Ni-O*	2.07±0.03	5.9±1.5	0.010±0.001	-4.1±0.9	169.3	0.00429
	Ni_2P	*Ni-P_1*	2.24±0.02	1.5±0.9	0.009±0.002			
	Ni_2P	*Ni-Ni*	2.56±0.01	1.3±0.2	0.009±0.002			
	$Ni(OH)_2$	*Ni-M_1*	3.08±0.01	3.6±0.7	0.009±0.002			
12h KOH NiP_x-FeO_x	$Ni(OH)_2$	*Ni-O*	2.05±0.01	7.0±0.4	0.007±0.001	-4.2±0.5	179.4	0.00272
	$Ni_2(PO_4)_3$	*Ni-P_2*	2.72±0.02	1.4±0.3	0.008±0.001			
	$Ni(OH)_2$	*Ni-M_1*	3.09±0.01	6.3±0.7	0.008±0.001			
**Sample**	**Path**	**Shell**	**R(Å)**	**CN**	**σ^2**	**E_o (eV)**	**χ^2**	**R-factor**
Pristine NiP_x	$Ni(OH)_2$	*Ni-O*	2.03±0.01	2.9±0.9	0.009±0.004	-6.8±1.6	663.2	0.00185
	Ni_2P	*Ni-P_1*	2.27±0.02	1.9±0.5	0.009±0.003			
	Ni_2P	*Ni-Ni*	2.56±0.01	1.5±0.6	0.009±0.003			
	$Ni(OH)_2$	*Ni-M_1*	3.07±0.01	1.6±0.5	0.009±0.003			
30CV NiP_x	NiOOH	*Ni-O_1*	1.92±0.02	3.1±0.5	0.005±0.002	-3.7±0.7	509.2	0.00949
	$Ni(OH)_2$	*Ni-O_2*	2.09±0.01	2.5±0.3	0.005±0.002			
	NiOOH	*Ni-M_1*	2.86±0.01	3.5±0.6	0.008±0.001			
	$Ni(OH)_2$	*Ni-M_2*	3.09±0.01	3.7±0.6	0.008±0.001			

Table S5. Fe K-edge EXAFS fit results for the pristine, 1, 5, 10, 20, 30 CV, and KOH-treated NiP_x-FeO_x nanocatalyst; and for the pristine and 30CV-treated FeO_x monometallic nanostructure.

Sample	Path	Shell	R(Å)	CN	σ^2	E_o (eV)	χ^2	R-factor
Pristine NiP_x-FeO_x	α-Fe_2O_3	*Fe-O*$_1$	1.92±0.04	2.8±1.0	0.003±0.004	-3.3±1.5	35.8	0.00202
	FeOOH	*Fe-O*$_2$	2.04±0.03	3.0±0.9	0.003±0.004			
	α-Fe_2O_3	*Fe-M*$_1$	2.94±0.04	1.6±0.8	0.003±0.005			
	FeOOH	*Fe-M*$_2$	3.09±0.03	2.4±0.9	0.003±0.005			
	α-Fe_2O_3	*Fe-M*$_3$	3.46±0.03	1.1±1.0	0.003±0.005			
1CV NiP_x-FeO_x	α-Fe_2O_3	*Fe-O*$_1$	1.92±0.02	3.2±0.5	0.003±0.004	-3.5±1.4	69.8	0.00189
	FeOOH	*Fe-O*$_2$	2.06±0.03	2.9±0.6	0.003±0.004			
	α-Fe_2O_3	*Fe-M*$_1$	2.93±0.03	1.5±1.0	0.005±0.006			
	FeOOH	*Fe-M*$_2$	3.09±0.03	3.3±1.4	0.005±0.006			
	α-Fe_2O_3	*Fe-M*$_3$	3.47±0.06	1.2±1.0	0.005±0.006			
5CV NiP_x-FeO_x	α-Fe_2O_3	*Fe-O*$_1$	1.94±0.03	3.4±1.0	0.003±0.003	-1.9±1.3	54.2	0.00291
	FeOOH	*Fe-O*$_2$	2.07±0.03	2.6±0.6	0.003±0.003			
	α-Fe_2O_3	*Fe-M*$_1$	2.95±0.05	1.8±1.0	0.009±0.001			
	FeOOH	*Fe-M*$_2$	3.10±0.03	5.2±0.9	0.009±0.001			
	α-Fe_2O_3	*Fe-M*$_3$	3.45±0.02	2.1±0.7	0.009±0.001			
10CV NiP_x-FeO_x	α-Fe_2O_3	*Fe-O*$_1$	1.95±0.05	3.7±0.7	0.007±0.006	-2.8±0.9	17.3	0.00275
	FeOOH	*Fe-O*$_2$	2.04±0.06	3.1±0.6	0.007±0.006			
	α-Fe_2O_3	*Fe-M*$_1$	2.94±0.07	1.5±0.9	0.009±0.007			
	FeOOH	*Fe-M*$_2$	3.10±0.02	5.2±1.5	0.009±0.007			
	α-Fe_2O_3	*Fe-M*$_3$	3.45±0.04	2.1±1.1	0.009±0.007			
20CV NiP_x-FeO_x	α-Fe_2O_3	*Fe-O*$_1$	1.94±0.04	3.0±0.5	0.005±0.004	-1.3±0.9	28.4	0.00377
	FeOOH	*Fe-O*$_2$	2.04±0.03	3.3±0.6	0.005±0.004			
	α-Fe_2O_3	*Fe-M*$_1$	2.93±0.03	1.8±0.8	0.008±0.006			
	FeOOH	*Fe-M*$_2$	3.10±0.02	5.2±4.0	0.008±0.006			
	α-Fe_2O_3	*Fe-M*$_3$	3.45±0.05	1.7±2.8	0.008±0.006			
30CV NiP_x-FeO_x	α-Fe_2O_3	*Fe-O*$_1$	1.95±0.07	3.0±0.8	0.005±0.006	-1.5±0.9	58.8	0.00321
	FeOOH	*Fe-O*$_2$	2.04±0.06	3.3±0.9	0.005±0.006			
	α-Fe_2O_3	*Fe-M*$_1$	2.91±0.04	1.7±0.7	0.009±0.010			
	FeOOH	*Fe-M*$_2$	3.10±0.02	6.6±0.9	0.009±0.010			
	α-Fe_2O_3	*Fe-M*$_3$	3.44±0.02	2.3±0.8	0.009±0.010			
12h KOH NiP_x-FeO_x	α-Fe_2O_3	*Fe-O*$_1$	1.92±0.06	2.5±1.1	0.004±0.006	-4.1±2.0	741.6	0.00220
	FeOOH	*Fe-O*$_2$	2.03±0.06	4.2±2.0	0.004±0.006			
	α-Fe_2O_3	*Fe-M*$_1$	2.89±0.06	1.6±0.9	0.010±0.013			
	FeOOH	*Fe-M*$_2$	3.09±0.03	7.0±2.5	0.010±0.013			
	α-Fe_2O_3	*Fe-M*$_3$	3.45±0.03	2.6±1.4	0.010±0.013			
**Sample**	**Path**	**Shell**	**R(Å)**	**CN**	σ^2	E_o **(eV)**	χ^2	**R-factor**
Pristine FeO_x	α-Fe_2O_3	*Fe-O*$_1$	1.91±0.02	2.2±0.4	0.004±0.002	2.6±1.0	542.3	0.00299
	FeOOH	*Fe-O*$_2$	2.06±0.02	1.8±0.3	0.004±0.002			
	α-Fe_2O_3	*Fe-M*$_1$	2.93±0.01	1.5±0.3	0.006±0.002			
	FeOOH	*Fe-M*$_2$	3.07±0.02	2.0±0.4	0.006±0.002			
	α-Fe_2O_3	*Fe-M*$_3$	3.49±0.01	2.4±0.8	0.006±0.002			
30CV FeO_x	α-Fe_2O_3	*Fe-O*$_1$	1.93±0.03	3.4±1.0	0.007±0.003	2.1±0.9	357.0	0.00090
	FeOOH	*Fe-O*$_2$	2.07±0.04	1.8±0.7	0.007±0.003			
	α-Fe_2O_3	*Fe-M*$_1$	2.92±0.03	1.5±0.5	0.007±0.002			
	FeOOH	*Fe-M*$_2$	3.05±0.02	2.7±0.4	0.007±0.002			
	α-Fe_2O_3	*Fe-M*$_3$	3.49±0.01	3.3±0.9	0.007±0.002			

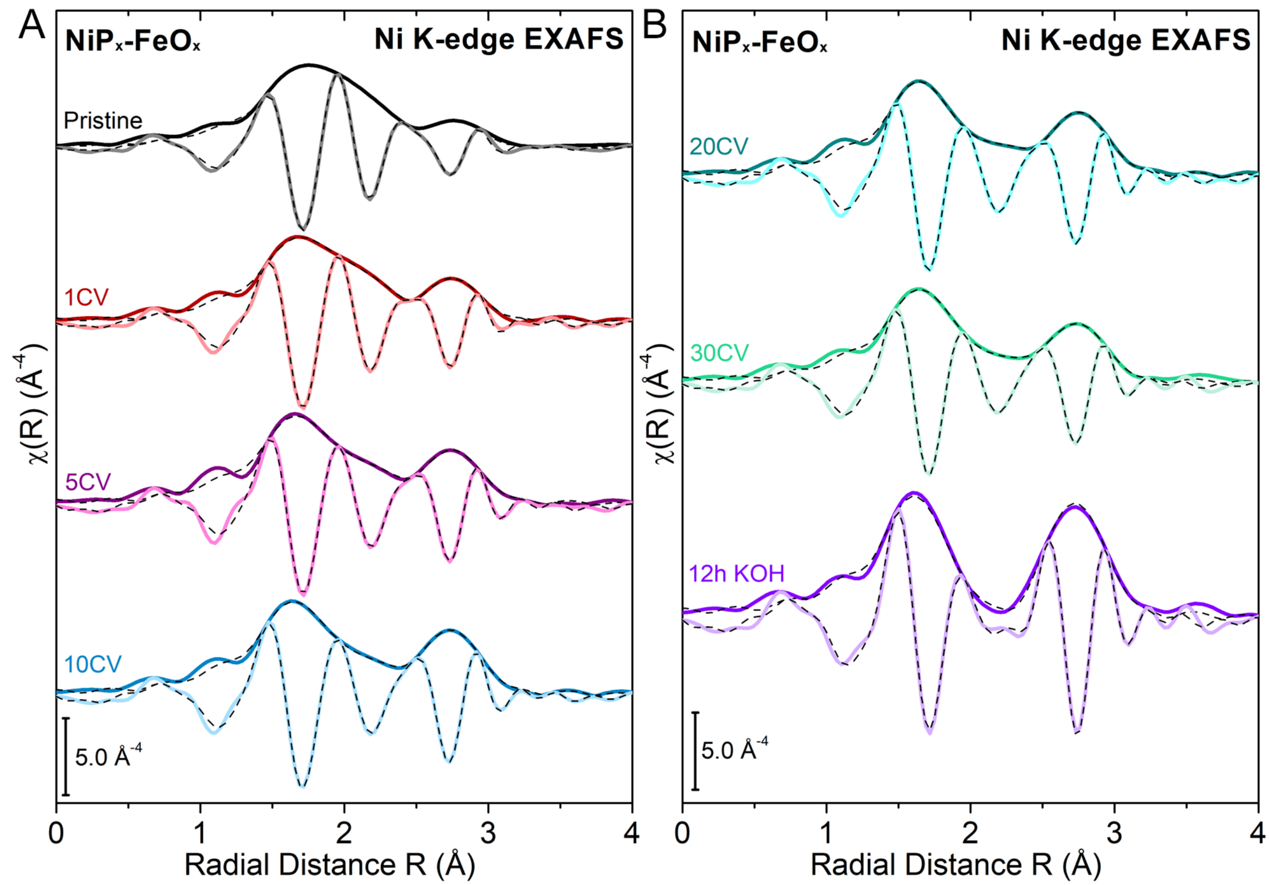

Figure S7. (A-B) The real (brightly colored) and imaginary (faded color) components of the Ni K-edge EXAFS results for the Pristine (black), 1 CV (red), 5 CV (Magenta), 10 CV (blue), 20 CV (cyan), 30 CV (green) treated, and 12 h KOH soaked (violet) NiP_x-FeO_x core-shell nanocatalyst with their respective fits (dotted line).

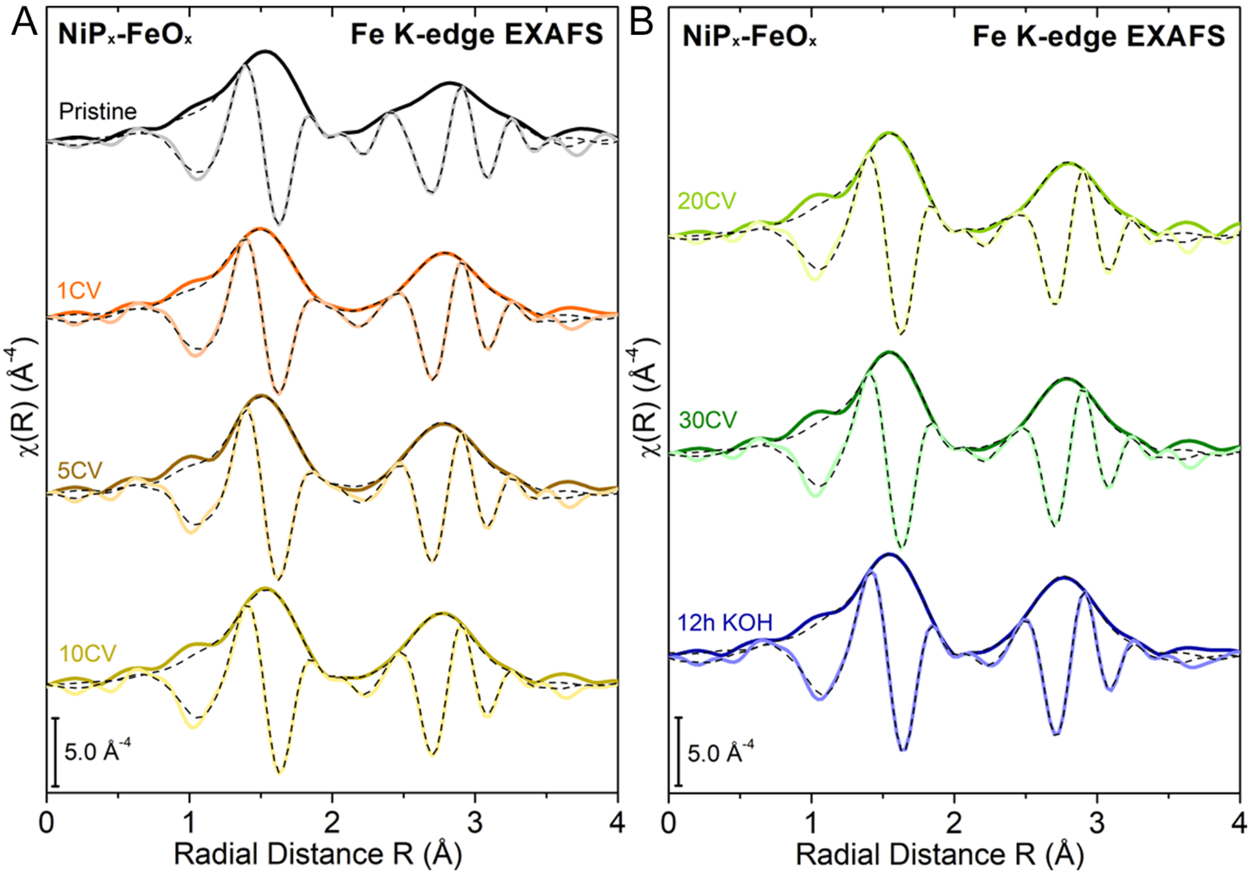

Figure S8. (A-B) The real (brightly colored) and imaginary (faded color) components of the Fe K-edge EXAFS results for the Pristine (black), 1 CV, (orange), 5 CV (brown), 10 CV (yellow), 20 CV, (light green), 30 CV (jade green) treated, and 12 h KOH soaked (purple) NiP_x-FeO_x core-shell nanocatalyst with their respective fits (dotted line).

Table S6. Average <Fe-O> bond lengths evaluated for the pristine, 1, 5, 10, 15, 20, 30 CV, and KOH-treated NiP_x-FeO_x nanocatalyst; and the pristine and 30 CV-treated monometallic FeO_x.

Sample/Treatment	Fe-O_1 (Å)	Fe-O_1 (CN)	Fe-O_2 (Å)	Fe-O_2 (CN)	Total CN	<Fe-O>
Pristine NiP_x-FeO_x	1.92±0.04	2.8±1.0	2.04±0.03	3.0±0.9	5.8±1.3	1.98±0.08
NiP_x-FeO_x 1CV	1.92±0.02	3.2±0.5	2.06±0.03	2.9±0.6	6.1±0.8	1.99±0.10
NiP_x-FeO_x 5CV	1.94±0.03	3.4±1.0	2.07±0.03	2.6±0.6	6.0±1.2	2.00±0.09
NiP_x-FeO_x 10CV	1.95±0.05	3.7±0.7	2.04±0.06	3.1±0.6	6.8±0.9	1.99±0.06
NiP_x-FeO_x 20CV	1.94±0.04	3.0±0.5	2.04±0.03	3.3±0.6	6.3±0.8	1.99±0.07
NiP_x-FeO_x 30CV	1.95±0.07	3.0±0.8	2.04±0.06	3.3±0.9	6.3±1.2	2.00±0.06
NiP_x-FeO_x 12h KOH	1.92±0.06	2.5±1.1	2.03±0.06	4.2±2.0	6.7±2.3	1.99±0.08
Pristine FeO_x	1.91±0.01	2.2±0.4	2.06±0.02	1.8±0.3	4.0±0.5	1.98±0.11
FeO_x 30CV	1.93±0.02	3.4±1.0	2.07±0.04	1.8±0.7	5.2±1.2	1.98±0.11

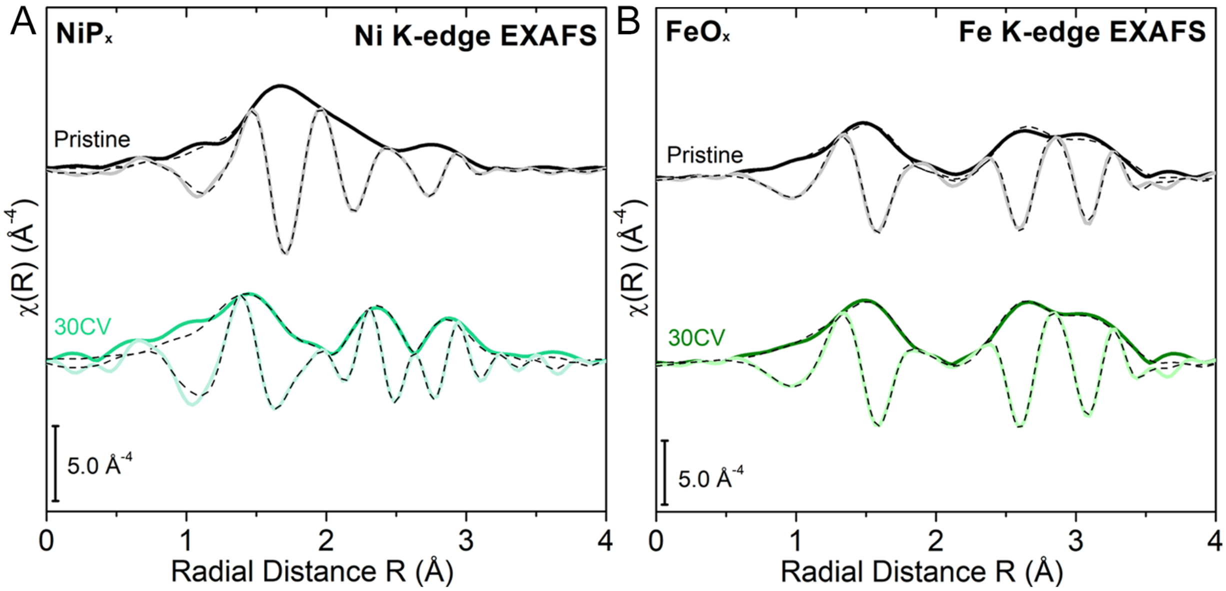

Figure S9. The real (brightly colored) and imaginary (faded color) components of the (A) Ni and (B) Fe K-edge EXAFS results for the Pristine (black) and 30 CV (blue) treated (A) NiP_x and (B) FeO_x monometallic nanoparticles with their respective fits (dotted line).

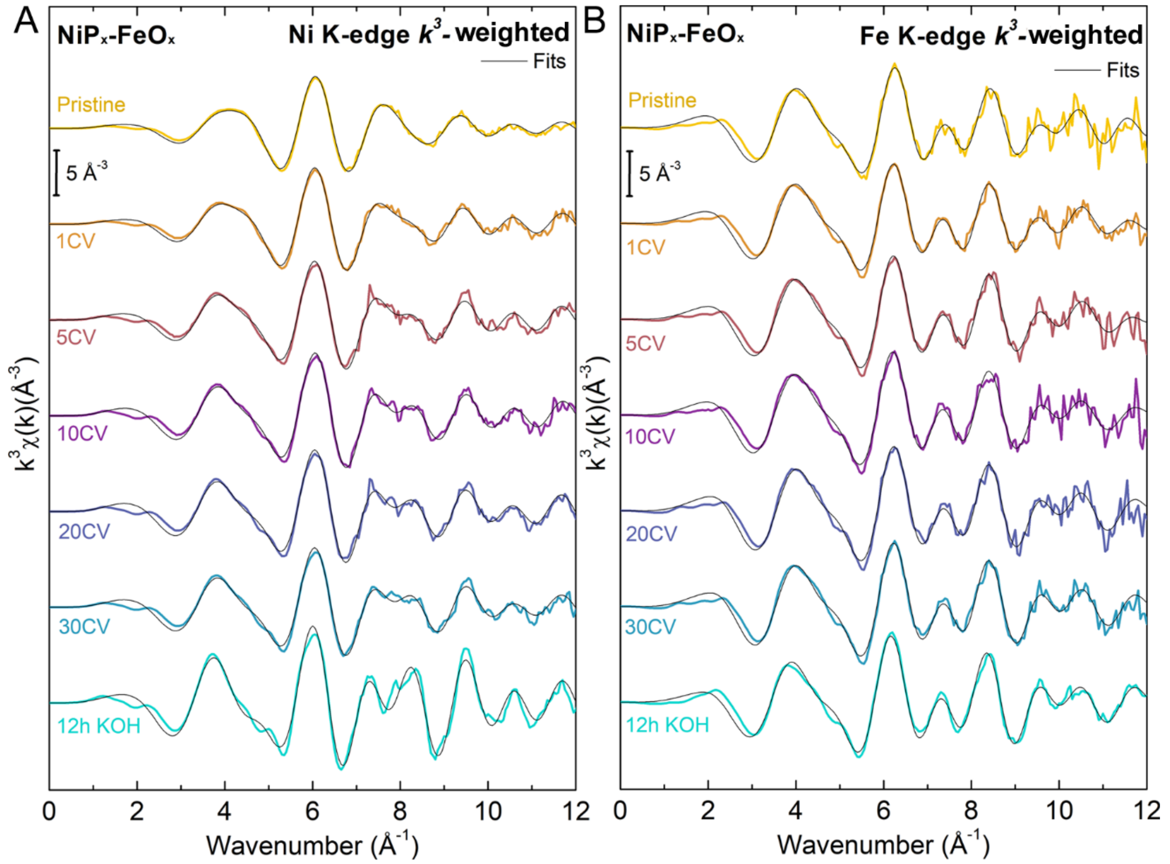

Figure S10. (A) Ni and (B) Fe K-edge k^3-weighted FT EXAFS spectra of the pristine NiP_x-FeO_x core-shell catalyst (yellow), and the 1 CV (orange), 5 CV (red), 10 CV (purple), 20 CV (royal blue), 30 CV (sky blue), and 12 h KOH (cyan) treated samples.

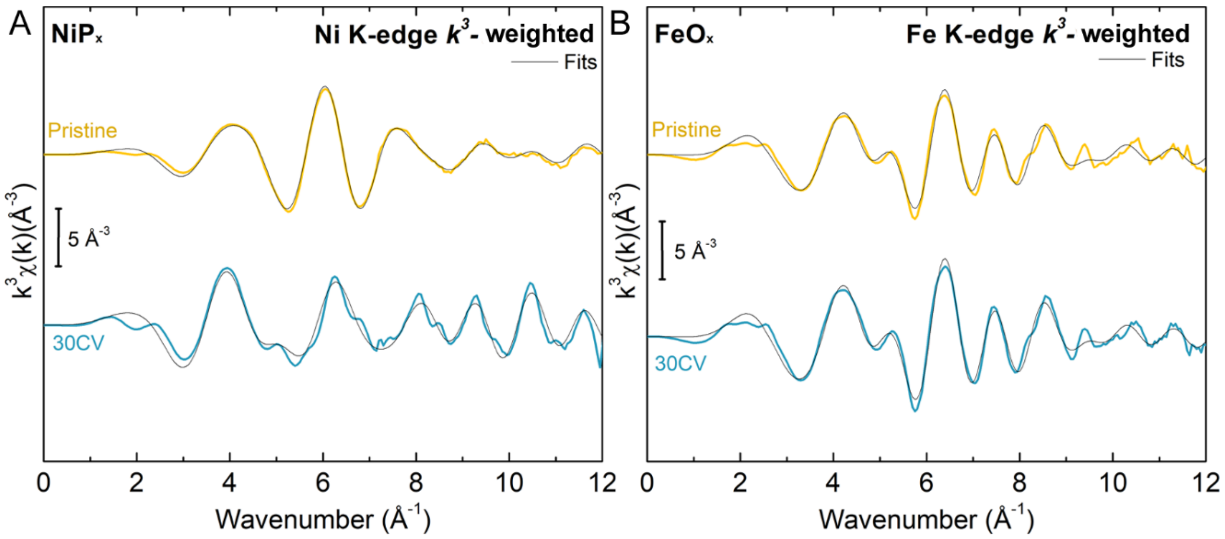

Figure S11. (A) Ni and (B) Fe K-edge k^3-weighted FT EXAFS spectra of the pristine NiP_x and FeO_x monometallic nanoparticles before (yellow) and after (blue) 30 CV treatment.

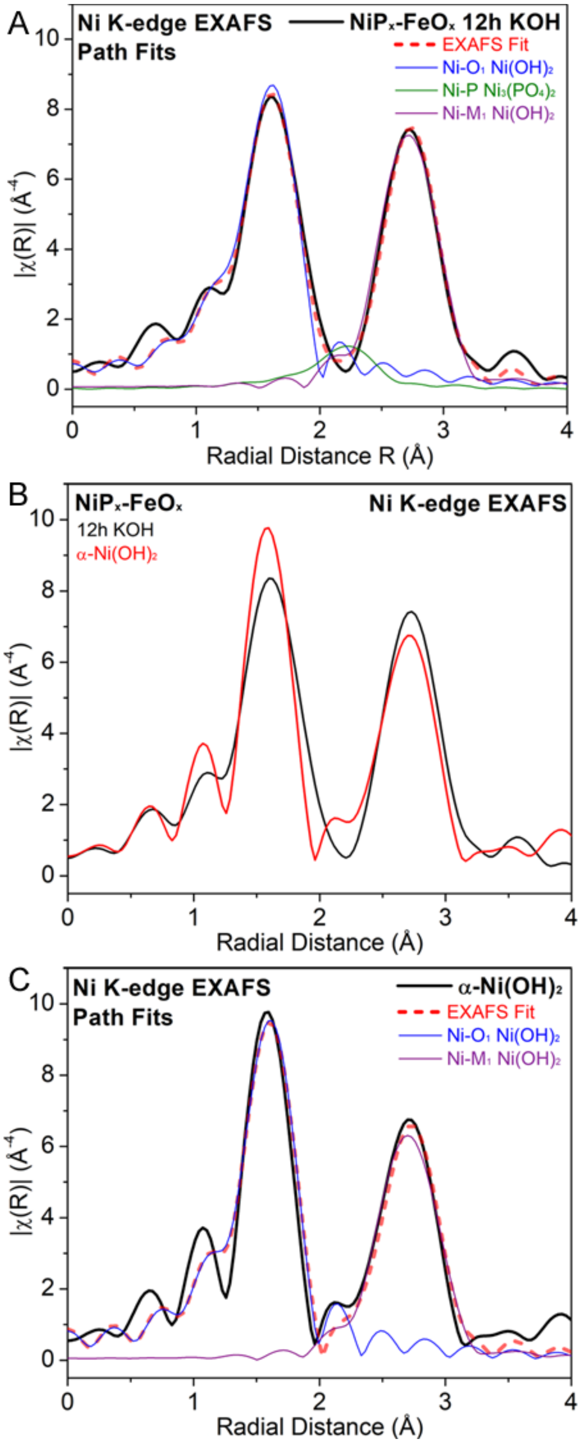

Figure S12. (A) Ni path contributions of the Ni K-edge FT EXAFS fit for 12h KOH treated NiP_x-FeO_x core-shell nanostructures; (B) α-$Ni(OH)_2$ vs. the 12 h KOH-treated NiP_x-FeO_x core-shell nanostructures; and (C) Ni path contributions of the Ni K-edge FT EXAFS fit for the α-$Ni(OH)_2$.

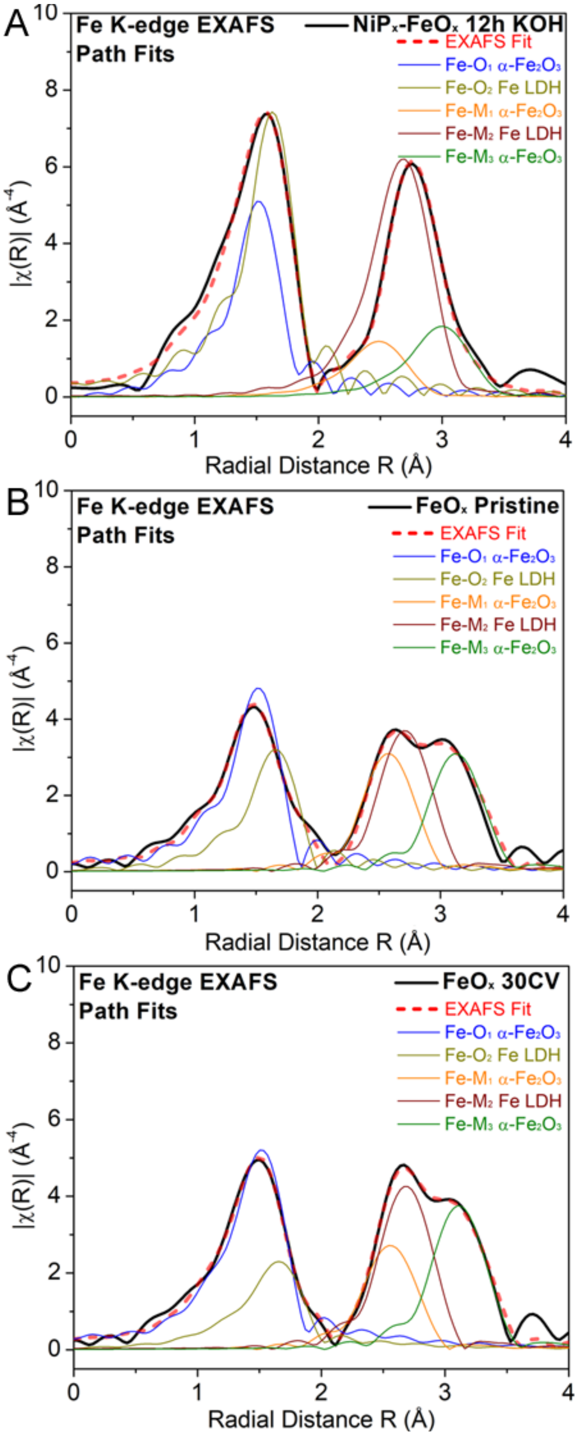

Figure S13. Fe path contributions of the Fe K-edge FT EXAFS fits: (A) 12 h KOH treated NiP_x-FeO_x core-shell nanostructures; (E,F) monometallic FeO_x nanoparticle before (E) and after 30 CV cycles (F).
